# Supplementary material for: Giant electrically tunable magnon transport anisotropy in a van der Waals antiferromagnetic insulator
Source: Nat Commun. 2023 May 2;14:2526. doi: 10.1038/s41467-023-38172-7 (PMC10154397; doi:10.1038/s41467-023-38172-7)
Supplement: Supplementary file 1 — Supplementary information [file 41467_2023_38172_MOESM1_ESM.pdf]

Supplementary information for
Giant electrically tunable magnon transport anisotropy in a van der Waals antiferromagnetic insulator

Shaomian Qi[1†], Di Chen[2†], Kangyao Chen[1], Jianqiao Liu[1], Guangyi Chen[1], Bingcheng Luo[1], Hang Cui[1], Linhao Jia[1,2], Jiankun Li[2], Miaoling Huang[2], Yuanjun Song[2], Shiyi Han[3], Lianming Tong[3], Peng Yu[4], Yi Liu[5], Hongyu Wu[6], Shiwei Wu[7], Jiang Xiao[7], Ryuichi Shindou[1], X.C. Xie[1,2], Jian-Hao Chen[1,2,8,9*]

[1]International Center of Quantum Materials, School of Physics, Peking University, Beijing, China

[2]Beijing Academy of Quantum Information Sciences, Beijing, China

[3]College of Chemistry and Molecular Engineering, Peking University, Beijing, China

[4]State Key Laboratory of Optoelectronic Materials and Technologies, School of Materials Science and Engineering, Sun Yat-sen University, Guangzhou, China

[5]Center for Advanced Quantum Studies and Department of Physics, Beijing Normal University, Beijing, China

[6]Key Laboratory of Magnetic Materials and Devices, Zhejiang Province Key Laboratory of Magnetic Materials and Application Technology, Ningbo Institute of Materials Technology and Engineering, Chinese Academy of Sciences, Ningbo, China

[7]Department of Physics and State Key Laboratory of Surface Physics, Fudan University, Shanghai, China

[8]Key Laboratory for the Physics and Chemistry of Nanodevices, Peking University, Beijing, China

[9]Interdisciplinary Institute of Light-Element Quantum Materials and Research Center for Light-Element Advanced Materials, Peking University, Beijing

[†]These authors contributed equally to this work.

[*]E-mail: Jian-Hao Chen (chenjianhao@pku.edu.cn)

Table of Contents:

S1. The insulating properties of $CrPS_4$ flake

S2. Determination of crystallographic orientation of $CrPS_4$

S3. Anisotropic magnon transport for more $CrPS_4$ devices

S4. The normalized $V_{2\omega,0}(I_{gate})/V_{2\omega,0}$ curves of the anisotropic magnon valves

S5. $V_{2\omega}$ vs. θ of a typical pair of $CrPS_4$ devices along <010> and <100> directions at various I_{gate}

S6. Local spin Seebeck measurements in $CrPS_4$ magnon valves

S7. Irrelevance of anomalous Nernst effect in $CrPS_4$ magnon valves

S8. Spin model of $CrPS_4$ under an in-plane magnetic field

S9. Modeling spin Seebeck effect in $CrPS_4$

S10. Ignorable out-of-plane spin Seebeck coefficient $S_z(T)$

S11. Finite element analysis of the temperature and spin chemical potential distribution in $CrPS_4$ device

S12. Simulated and experimental $V^S_{2\omega,0}$ and $V^W_{2\omega,0}$ vs. I_{gate} curves

S13. Simulation analysis for anisotropic magnon transport tuning

S14. Stability test of few-layer $CrPS_4$ device

S15. Possible applications of the magnon ROM

S1. The insulating properties of $CrPS_4$ flake

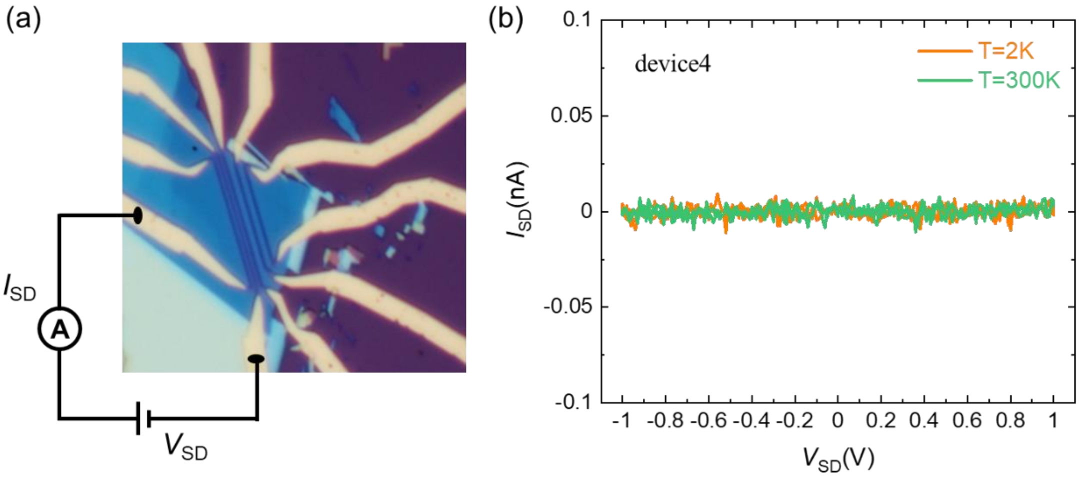

Fig. S1. The insulating properties of $CrPS_4$ flake. (a) Schematic of the two-probe measurement geometry on the $CrPS_4$ device. (b) The current vs. voltage curves between two parallel Pt electrodes across 750 nm $CrPS_4$ channel at T = 2 K and 300 K.

S2. Determination of crystallographic orientation of $CrPS_4$

To study the anisotropic magnon transport, it is necessary to fabricate the two magnon valve devices strictly along the <100> and <010> crystallographic directions. The achievement of such alignment is ensured in the following two ways. First of all, both transmission electron microscopy and polarized optical microscopy have shown that the exfoliated $CrPS_4$ samples often cleave along the <110> direction, i.e., along the diagonal Cr atom rows with respect to the <100> direction and <010> direction, forming an acute angle of 67.5° in between[1,2]. The existence of such easy cleavage lines allows ones to readily identify the crystallographic orientations of $CrPS_4$ samples optically as annotated in Fig. 1b in the main text. As expected, our exfoliated $CrPS_4$ samples were terminated with straight edges that formed characteristic angle 67.5° as shown in Fig. 1d in the main text, so that the diagonal line of the angle 67.5° is the <100> direction.

The second method to determine the crystal orientation is angle-dependent linearly polarized Raman spectroscopy[3, 4]. In the particular case of $CrPS_4$ thin flakes, the Raman A_2 mode at 169.0 cm^{-1} (when excited by a laser of 514 nm and 633 nm in wavelength) has a two-fold angular dependence in the intensity pointing along the <010> direction[4]. We acquired such Raman data after the fabrication of the $CrPS_4$ devices and confirmed the accurate alignment of our devices with the respective crystal axes. As illustrated in Fig. S2a, Raman scattering measurements were performed using 532 nm laser excitation in a back-scattering micro-Raman configuration with HR800 (Jobin Yvon Horiba). Polarized measurements in the parallel and cross configurations are obtained by adjusting the relative angle between polarizer2 at the scattered light path and polarizer1 at the incident light path. To vary the azimuthal angle, we rotated the sample in a step of 10° using a rotational mount. We define the angle between the x axis and the incident beam polarization vector $\mathbf{e_i}$ as θ shown in Fig. S2b.

$CrPS_4$ belongs to C_2 point group with one unit cell containing $Cr_2P_2S_8$. The lattice vibrations at the Brillouin zone center consist of the following irreducible representations: $17A + 19B$, in which all the optical modes ($16A+17B$) are Raman-active[4]. As shown in the polarized angle-dependent spectra for the region of 100-600 cm^{-1} (Fig. S2c,d), the intensity of each Raman peak varied substantially as a function of θ, which reveals the lattice symmetry. According to the polarization selection rules, the angle-dependent Raman mode intensity is represented as:

$$I \propto \left|\mathbf{e_i} \cdot \widetilde{\mathbf{R}} \cdot \mathbf{e_s}\right|^2 \quad \text{(S1)}$$

where $\mathbf{e_i}$ and $\mathbf{e_s}$ are the polarization vectors for the incident light and scattered light, respectively, and $\widetilde{\mathbf{R}}$ is the Raman tensor of a given mode. The Raman tensors corresponding to C_2 symmetry group are expressed as:

$$\widetilde{\mathbf{R}}(A) = \begin{pmatrix} be^{i\Phi_b} & 0 & de^{i\Phi_d} \\ 0 & ce^{i\Phi_c} & 0 \\ de^{i\Phi_d} & 0 & ae^{i\Phi_a} \end{pmatrix}, \quad \widetilde{\mathbf{R}}(B) = \begin{pmatrix} 0 & fe^{i\Phi_f} & 0 \\ fe^{i\Phi_f} & 0 & ee^{i\Phi_e} \\ 0 & ee^{i\Phi_e} & 0 \end{pmatrix} \quad \text{(S2)}$$

By substituting the polarization vectors with parallel and cross configurations as well as the Raman tensors into equation S1, the following expressions can be obtained for the Raman intensities in the parallel (||) and cross (⊥) configuration for both types of Raman peaks:

$$I_A^{\parallel} \propto b^2 sin^4\theta + c^2 cos^4\theta + 2bc sin^2\theta cos^2\theta cos\Phi_{bc} \quad \text{(S3)}$$

$$I_A^{\perp} \propto sin^2\theta cos^2\theta (b^2 + c^2 - 2bc cos\Phi_{bc}) \quad \text{(S4)}$$

$$I_B^{\parallel} \propto (f\, sin\, 2\theta)^2 \quad \text{(S5)}$$

$$I_B^{\perp} \propto (f\, cos\, 2\theta)^2 \quad \text{(S6)}$$

where Φ_{bc} is the difference between Φ_b and Φ_c. It is straightforward that B modes have a four-fold symmetry with maxima at 0° and 90° in cross polarization configuration, which gives <100> and <010> axes. Particularly, A modes have a two-fold symmetry in parallel polarization configuration, which helps us to distinguish <100> and <010> axes. Fig. S2e and S2f display the polar graphs of peak intensities $I_B^{\perp}$ for B_5 mode (256.6cm^{-1}) and $I_A^{\parallel}$ for A_2 mode (169.0cm^{-1}) as a function of the azimuthal angle θ, respectively. The A_2 mode have a two-fold symmetry with maxima at 90° in parallel polarization configuration, implying that the x axis we defined is the <100> direction, which is consistent with previous report[4].

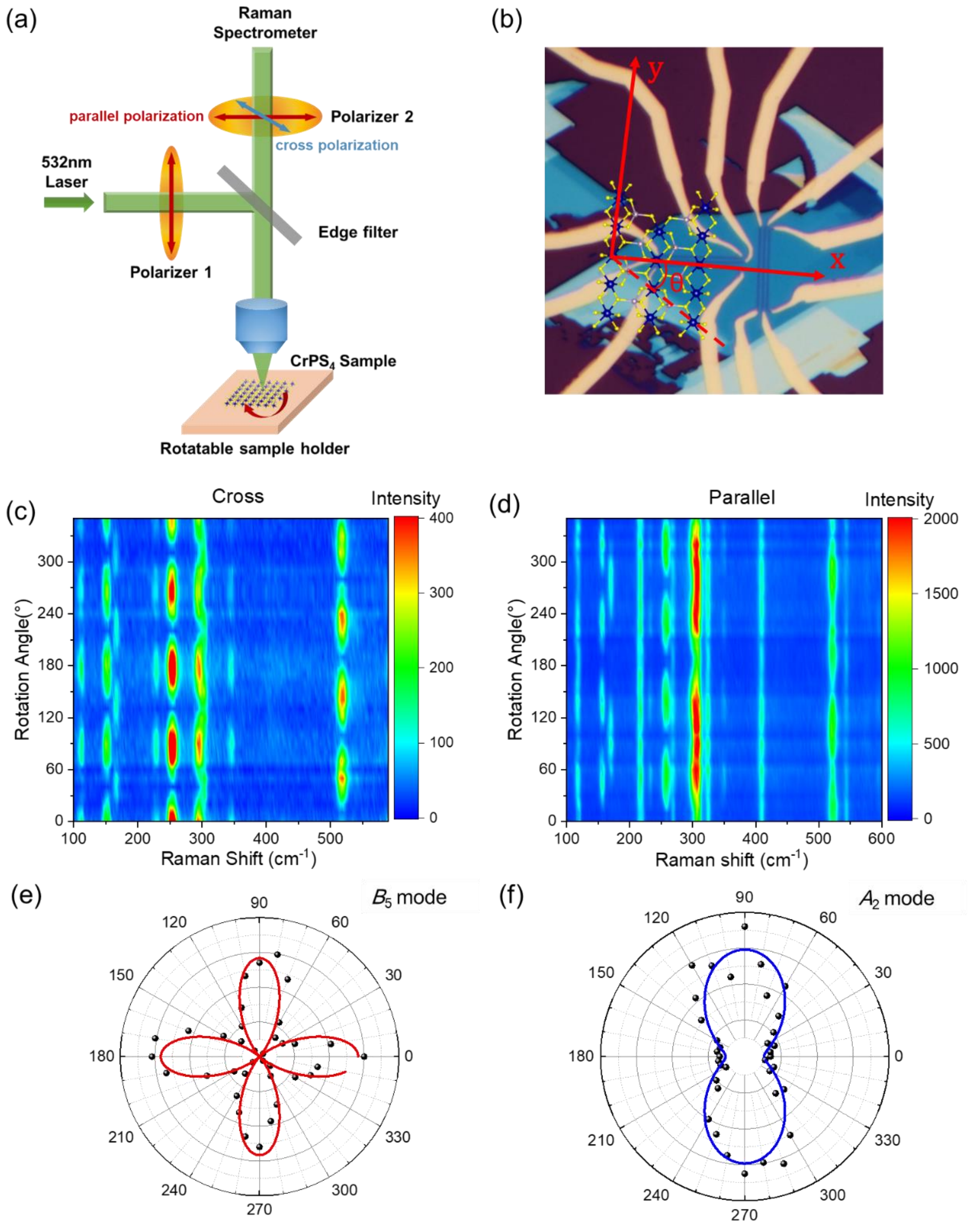

Fig. S2. Angle-dependent Raman spectroscopy of a $CrPS_4$ device. (a) Schematic of the angle-dependent polarized Raman scattering measurement. (b) Optical image of a typical $CrPS_4$ device and the definition of azimuthal angle θ. (c) Angle-dependent Raman spectra intensity plot under cross polarization configuration. (d) Angle-dependent Raman spectra intensity plot under parallel polarization configuration. (e) Polar graph of cross Raman signals of B_5 mode. Red solid line is a fit to equation S6. (f) Polar graph of parallel Raman signals of A_2 mode. Blue solid

line is a fit to equation S3.

S3. Anisotropic magnon transport for more $CrPS_4$ devices

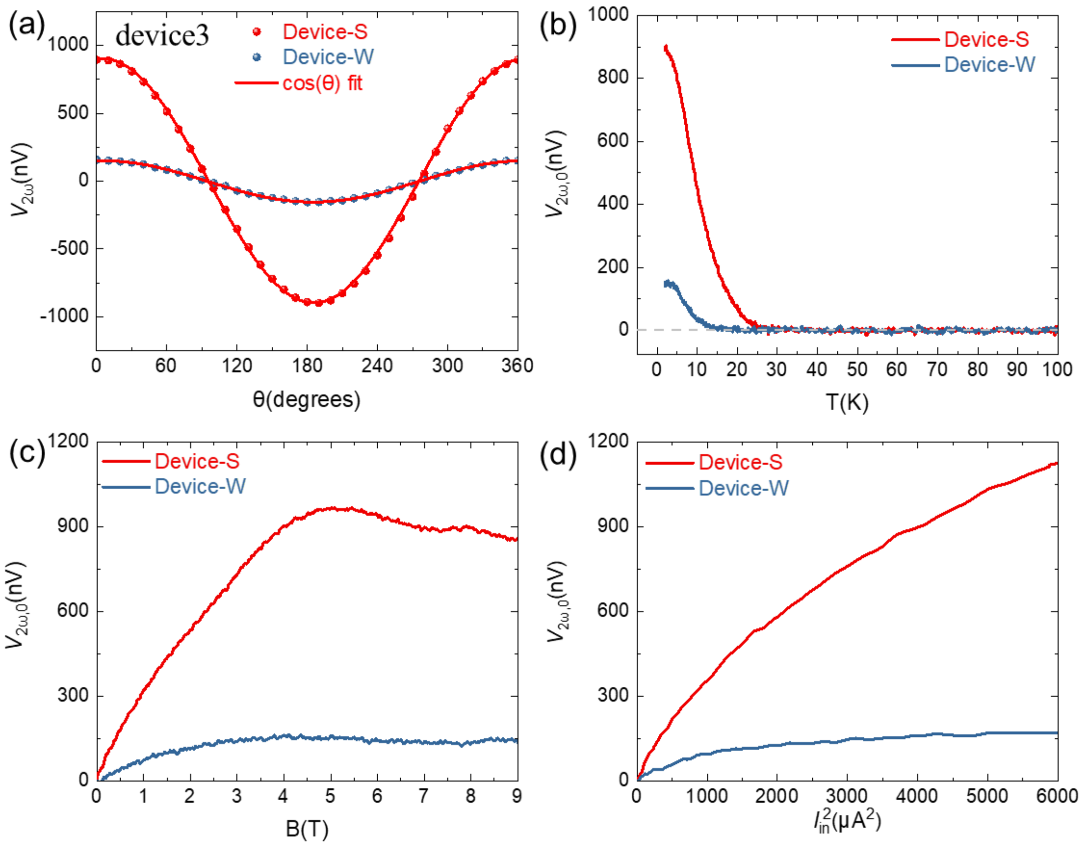

Fig. S3.1. Anisotropic magnon transport of another $CrPS_4$ device with channel length of 1.5 μm. (a) $V_{2\omega}$ as a function of the angle θ between the external magnetic field **B** and the direction perpendicular to Pt bars. The solid lines are fittings to cosine function. (b) Temperature dependence of $V_{2\omega}$ at $\theta = 0$ ($V_{2\omega,0}$). (c) Magnetic field dependence of $V_{2\omega,0}$. (d) $V_{2\omega,0}$ versus the square of the injection current I_{in}^2. Red and blue curves show magnon transport signals taken along the <010> (Device-S) and <100> (Device-W) directions of the $CrPS_4$ crystal, respectively.

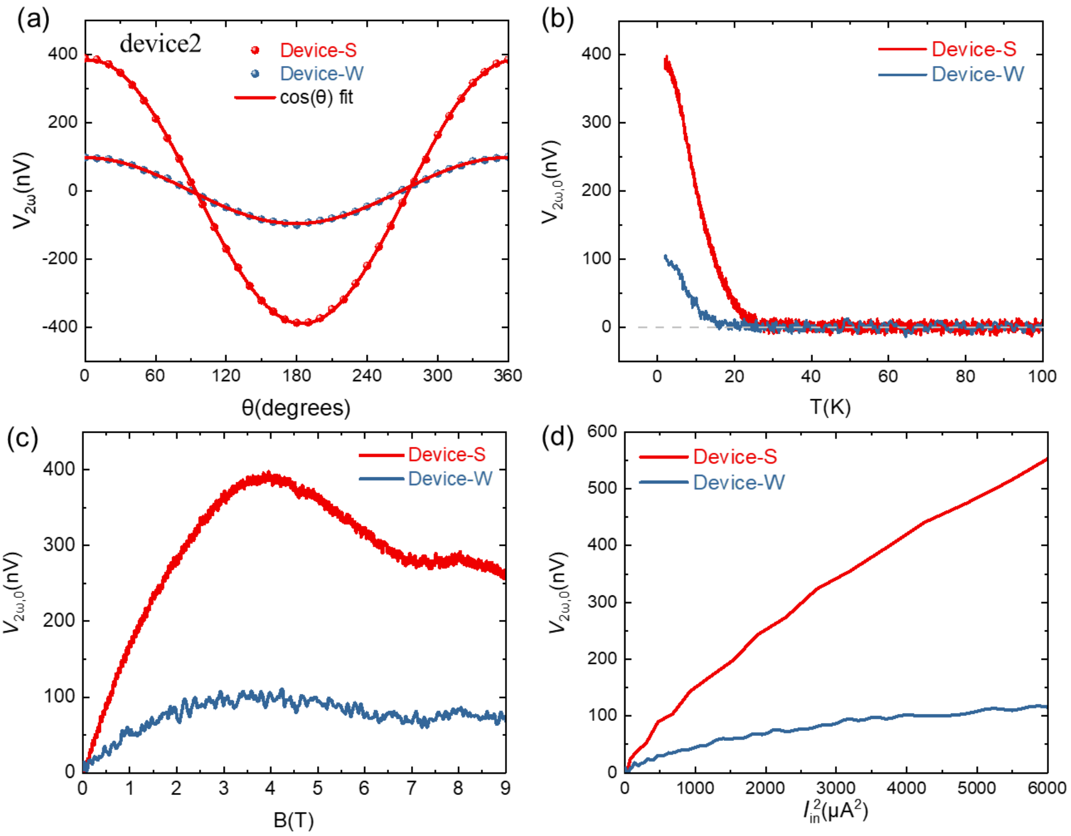

Fig. S3.2. Anisotropic magnon transport of another $CrPS_4$ device with channel length of 1.5 μm. (a) $V_{2\omega}$ as a function of the angle θ between the external magnetic field **B** and the direction perpendicular to Pt bars. The solid lines are fittings to cosine function. (b) Temperature dependence of $V_{2\omega}$ at $\theta = 0$ ($V_{2\omega,0}$). (c) Magnetic field dependence of $V_{2\omega,0}$. (d) $V_{2\omega,0}$ versus the square of the injection current I_{in}^2. Red and blue curves show magnon transport signals taken along the <010> (Device-S) and <100> (Device-W) directions of the $CrPS_4$ crystal, respectively.

S4. The normalized $V_{2\omega,0}(I_{gate})/V_{2\omega,0}$ curves of the anisotropic magnon valves

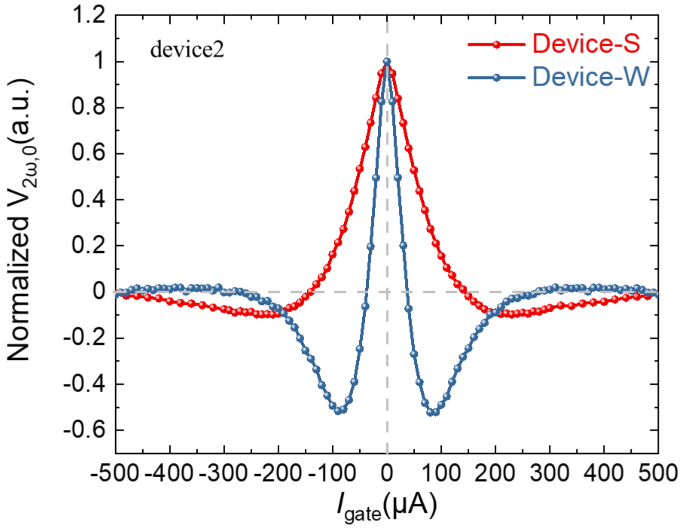

Fig. S4. Normalized I_{gate} dependent $V_{2\omega,0}$ curves of the data shown in Fig. 3a of the main text.

S5. $V_{2\omega}$ vs. θ of a typical pair of $CrPS_4$ devices along <010> and <100> directions at various I_{gate}

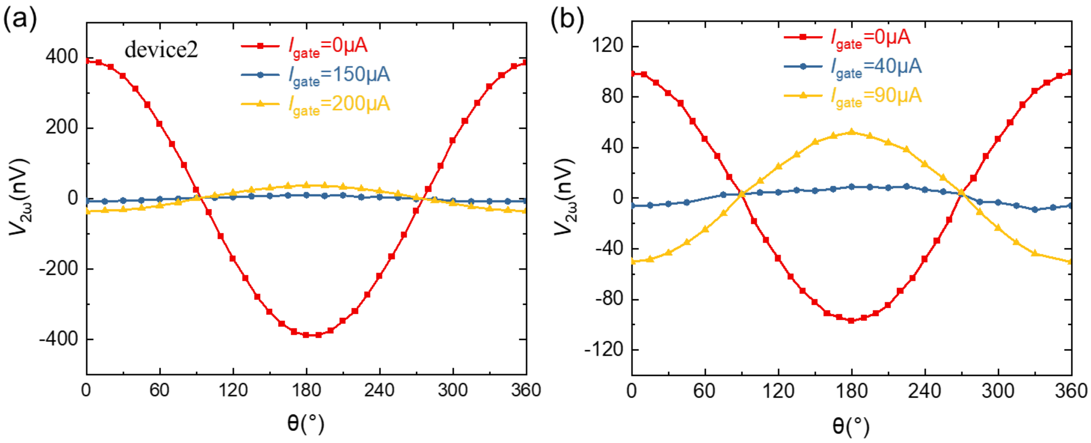

Fig. S5. Magnetic field angle dependent $V_{2\omega}$ of a typical $CrPS_4$ device at various I_{gate}. (a) $V_{2\omega}^{S}$ for Device-S as a function of angle θ of the external magnetic field at $I_{gate} = 0,150,200$μA. (b) $V_{2\omega}^{W}$ for Device-W as a function of angle θ of the external magnetic field at $I_{gate} = 0,40,90$μA. The $cos\theta$ dependence of the curves confirms the thermal magnon nature of the signal.

S6. Local spin Seebeck measurements in $CrPS_4$ magnon valves

Here, the definition of "local spin Seebeck effect" in our nonlocal measurement configuration is two-fold: 1) nonlocal transport of phonons in the $CrPS_4$ channel instead of magnons; 2) the nonlocal phonons created temperature gradient at the detector-$CrPS_4$ interface and produced second harmonic signal via local spin Seebeck effect.

To eliminate the possibility of such local spin Seebeck effect in our nonlocal measurement configuration, we measured the local spin Seebeck signal of the injector electrode $V_{2\omega,local}$ as a function of I_{gate} through the gate electrode next to the injector electrode. As shown in Fig. S6a-b, the $V_{2\omega,local}(I_{gate})$ curves with magnetic field direction $\theta = 0°$ and $180°$ for Device-S and Device-W under the same experimental conditions as in our nonlocal measurements. The $V_{2\omega,local}$ signals have constant offsets, thus we can define the pure local spin Seebeck signal without offset to be:

$$V_{2\omega,pure} = \left[V_{2\omega,local}\left(I_{gate}, \theta = 180°\right) - V_{2\omega,local}\left(I_{gate}, \theta = 0°\right)\right]/2$$

and the offset voltage to be:

$$V_{offset} = \left[V_{2\omega,local}\left(I_{gate}, \theta = 180°\right) + V_{2\omega,local}\left(I_{gate}, \theta = 0°\right)\right]/2$$

which are shown in Fig. S6c-d. Device-S and Device-W have very similar $V_{offset}(I_{gate})$ curves that are independent of magnetic field directions and crystallographic directions, which indicate an ordinary Seebeck effect from the Pt electrodes.

In order to verify the spin Seebeck effect nature of the $V_{2\omega,pure}$ and the non-magnetic nature of V_{offset}, magnetic field angle dependence of the raw data, i.e., $V_{2\omega,local}\left(I_{gate}\right) vs. \theta$ is shown in Fig. S6e-f. Here Fig. S6e shows the $V_{2\omega,local}\left(I_{gate}\right) vs. \theta$ curves for a set of discrete I_{gate} values for the strong exchange coupling direction, whereas Fig. S6f shows similar data for the weak

exchange coupling direction. The curves for different I_{gate} are all fitted to $-V_{2\omega,pure}\cos(\theta)+V_{offset}$ where $V_{2\omega,pure}$ and V_{offset} are fitting parameters. The fitted parameters are then plotted together with the data in Fig. S6c-d, which are quite consistent with the anti-symmetric and symmetric components extracted from $V_{2\omega.local}(I_{gate})$ curves.

Comparing Fig. S6c with features of the nonlocal $V_{2\omega,0}(I_{gate})$ curves exhibited in Fig. 3a in the main text, we can see that the two effects are completely different: 1) When $I_{gate}=0$, the local $V_{2\omega,pure}^{S}$ for Device-S is only 1.8 times of the local $V_{2\omega,pure}^{W}$ of Device-W, while for the nonlocal data, such ratio reaches to 4.0 times; 2) I_{gate} turns the nonlocal signals down for both Device-S and Device-W, however the local signals don't go to zero and show no inverse sign change like the nonlocal signals. These features unambiguously show that the magnon diffusive process and anisotropic magnetic exchange interactions are vital in producing the highly tunable anisotropic nonlocal signal we reported in the main text.

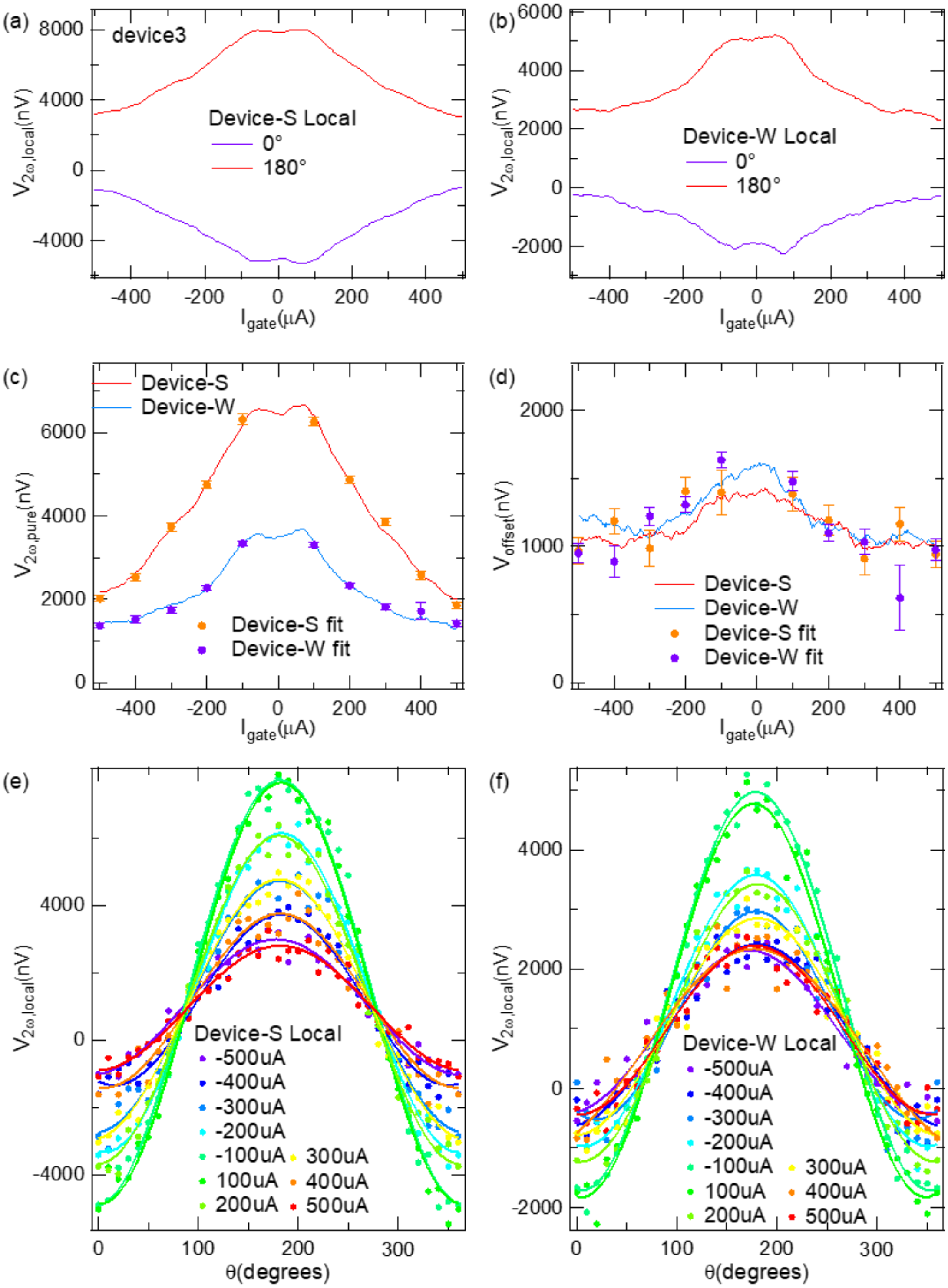

Fig. S6. Local spin Seebeck measurements in CrPS₄ magnon valves. (a) Local $V_{2\omega,local}$ vs. I_{gate} curves for Device-S (Pt electrode perpendicular to the <010> direction) and (b) Device-W (Pt electrode perpendicular to the <100> direction) at $\mathbf{B} = 4\text{T}$, $I_{in} = 60\ \mu A$ and $T = 2$ K. Purple and red curves show signals taken under $\theta = 0°$ and $180°$ field directions, respectively. (c) Spin Seebeck signal $V_{2\omega,pure}$ vs.

I_{gate} curves and (d) field direction irrelevant V_{offset} vs. I_{gate} curves for Device-S (red curves) and Device-W (blue curves). Orange and purple dots are obtained from fitting curves in (e) and (f), and the error bar denotes the standard deviation of the fitting. (e) $V_{2\omega,local}$ vs. angle θ between the external B field and the direction perpendicular to respective Pt electrodes for Device-S and (f) Device-W with different I_{gate}. The solid curves are fits to a cosine function plus a constant.

S7. Irrelevance of the anomalous Nernst effect in $CrPS_4$ magnon valves

$CrPS_4$ is an antiferromagnetic material with intralayer spins ferromagnetically aligned and interlayer spins antiferromagnetically aligned, which means the topmost layer contact with Pt is ferromagnetic. In order to quantify the possible effect of the anomalous Nernst effect in our $CrPS_4$ devices, we have measured the non-local second harmonic signal with an applied magnetic field of up to 9 T rotated in the *x*-*z* plane (see inset in Fig. S7 below). The angle of the magnetic field with respect to the *z* axis is marked as φ. An injection current of 60 μA is applied to the injector of our $CrPS_4$ device. We can see from Fig. S7a that the data fits well to a sinφ function, in which the signal is zero when the magnetic field is along the *z* axis (perpendicular to the sample plane) and only the magnetic field component along the *x* axis could produce non-zero non-local second harmonic signal. Fig. S7b shows minimal magnetic-field dependence of the non-local signal with the magnetic field along the *z* axis (i.e., φ=0). This data proves unambiguously the absence of anomalous Nernst effect with magnetic field perpendicular to the $CrPS_4$/Pt interface (ANE_x). It's considered that ANE_x and ANE_z are of similar magnitude[5], where ANE_z refers to the Hall voltage along y induced by the temperature gradient along *z* in the presence of the magnetization along the *x* axis. Thus, the absence of ANE_x points to the absence of ANE_z in our $CrPS_4$ magnon devices, pointing to the irrelevance of anomalous Nernst effect in the electrically tunable anisotropic SHM response.

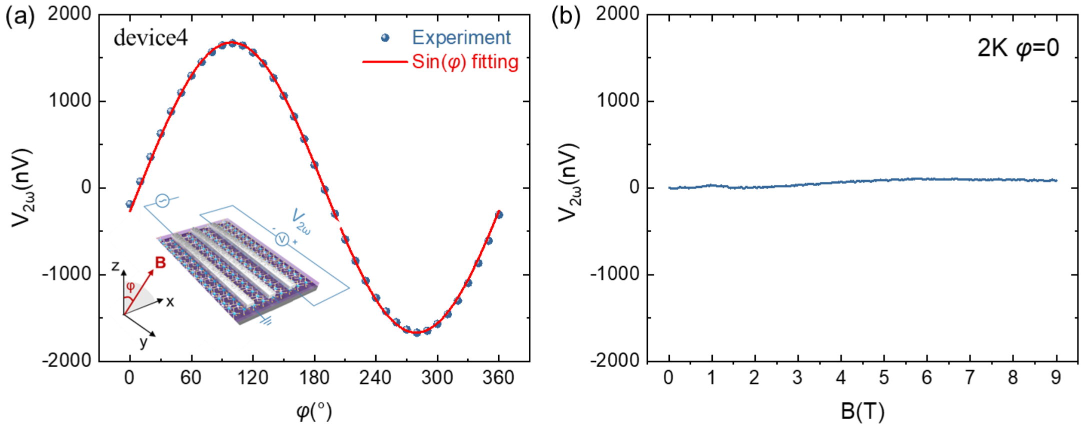

Fig. S7. Irrelevance of the anomalous Nernst effect in CrPS4 device. (a) The non-local second harmonic signal as a function of angle φ between the external magnetic field (**B**=9T) and the *z* direction, angle φ is defined as shown in the inset. (b) Very little magnetic field dependence can be observed for $V_{2\omega}$ at $\varphi = 0$, showing that anomalous Nernst effect, if exist, does not interfere with our analysis.

S8. Spin model of CrPS4 under an in-plane magnetic field

In this section, we consider a two-dimensional localized spin model with easy-axis single-ion anisotropy containing bilayer atoms to describe the A-type antiferromagnet CrPS4. We carry out spin-wave analysis of the antiferromagnet under a transverse magnetic field to obtain low-energy magnon excitations.

Magnetism for the bilayer CrPS4 is described by spin model:

$$H = H_1 + H_2 + H_{1,2} \tag{S7}$$

$$H_1 = \sum_{\boldsymbol{j}} \sum_{m=1,2,3,4} J_{\boldsymbol{a_m}} \boldsymbol{S}_{1,\boldsymbol{j}}^{A} \cdot \boldsymbol{S}_{1,\boldsymbol{j}+\boldsymbol{a_m}}^{B} - D \sum_{\boldsymbol{j}} \left[\left(S_{1,\boldsymbol{j}}^{A,z}\right)^2 + \left(S_{1,\boldsymbol{j}}^{B,z}\right)^2 \right] - h \sum_{\boldsymbol{j}} \left[S_{1,\boldsymbol{j}}^{A,y} + S_{1,\boldsymbol{j}}^{B,y} \right]$$

$$H_2 = \sum_{\boldsymbol{j}} \sum_{m=1,2,3,4} J_{\boldsymbol{a_m}} \boldsymbol{S}_{2,\boldsymbol{j}}^{A} \cdot \boldsymbol{S}_{2,\boldsymbol{j}+\boldsymbol{a_m}}^{B} - D \sum_{\boldsymbol{j}} \left[\left(S_{2,\boldsymbol{j}}^{A,z}\right)^2 + \left(S_{2,\boldsymbol{j}}^{B,z}\right)^2 \right] - h \sum_{\boldsymbol{j}} \left[S_{2,\boldsymbol{j}}^{A,y} + S_{2,\boldsymbol{j}}^{B,y} \right]$$

$$H_{1,2} = J_c \left[\sum_{\boldsymbol{j}} \boldsymbol{S}_{1,\boldsymbol{j}}^{A} \cdot \boldsymbol{S}_{2,\boldsymbol{j}}^{A} + \boldsymbol{S}_{1,\boldsymbol{j}}^{B} \cdot \boldsymbol{S}_{2,\boldsymbol{j}}^{B} \right]$$

Here H_1 is Hamiltonian for the first layer, H_2 is Hamiltonian for the second layer, $H_{1,2}$ describes the interlayer interaction term. D is the easy-axis single-ion

anisotropy and J is the magnetic exchange coupling with $J_{a_1}=J_1$, $J_{a_2}=J_2$, $J_{a_3}=J_3$, $J_{a_4}=J_3$. The parameters (J_1 to J_4) we used are shown in Fig. S8.1, which are reported by S. Calder *et al.* from neutron scattering measurements[6]. Subscripts A,B represent two sets of sublattices of $CrPS_4$, $\boldsymbol{j}$ denotes a monoclinic-lattice A-sublattice site, and $\mathbf{a}_m$ $(m=1,2,3,4)$ connects a A-sublattice site and its neighboring four B-sublattice site with $\mathbf{a}_1=0$, $\mathbf{a}_2=\mathbf{e}_2, \mathbf{a}_3=\mathbf{e}_1, \mathbf{a}_4=\mathbf{e}_2-\mathbf{e}_1$. $\mathbf{S}_j^{\mathrm{A}} \equiv \left(S_{\mathbf{j}}^{\mathrm{A,x}}, S_{\mathbf{j}}^{\mathrm{A,y}}, S_{\mathbf{j}}^{\mathrm{A,z}}\right)$ is a localized spin of Cr atom (S=3/2) in the A-sublattice site $(\mathbf{j})$ and $\mathbf{S}_{\mathbf{j}+\mathbf{a}_m}^{\mathrm{B}}$ is a localized Cr spin at the B-sublattice site$(\mathbf{j}+\mathbf{a}_m)$. For simplicity, we consider that the system is a two-dimensional magnet and h is an in-plane field along $x(y)$-direction for Device-W (Device-S).

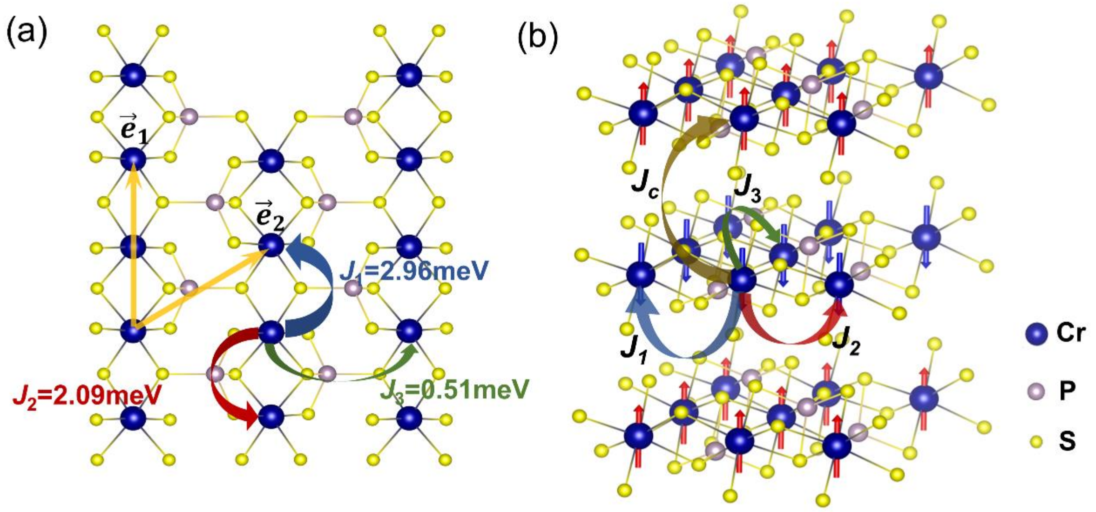

J_1	J_2	J_3	J_c	D_z
-2.96meV	-2.09meV	-0.51meV	0.16meV	0.0058meV

Fig. S8.1. Magnetic structure parameters of $CrPS_4$[6].

Under the in-plane field, the antiferromagnetic moment will be deformed linearly in the field:

$$\boldsymbol{S}_{1,\boldsymbol{j}}^{A}=\boldsymbol{S}_{1,\boldsymbol{j}}^{B}=S(0, sin\psi, \cos\psi)$$

$$\boldsymbol{S}_{2,\boldsymbol{j}}^{A}=\boldsymbol{S}_{2,\boldsymbol{j}}^{B}=S(0, sin\psi, -\cos\psi)$$

A canting angle is determined classically as a minimum of a classical magnetic energy:

$$E_{classical} = 2N(J_c S^2(\sin^2\psi - \cos^2\psi) - 2DS^2\cos^2\psi - 2hS\sin\psi),$$

where N is a number of the A-sublattice sites. The minimum energy is given by:

$$\sin\psi = \frac{h}{2(2J_c + D)S} \tag{S8}$$

Magnetic collective excitations around the classical magnetic order are described by Holstein-Primakoff bosons:

$$\tilde{S}^{A,z}_{1,j} = S - a^\dagger_{1,j}a_{1,j}, \quad \tilde{S}^{A,x}_{1,j} - i\tilde{S}^{A,y}_{1,j} = \sqrt{2S}a^\dagger_{1,j}, \quad \tilde{S}^{A,x}_{1,j} + i\tilde{S}^{A,y}_{1,j} = \sqrt{2S}a_{1,j}$$

$$\tilde{S}^{B,z}_{1,j} = S - b^\dagger_{1,j}b_{1,j}, \quad \tilde{S}^{B,x}_{1,j} - i\tilde{S}^{B,y}_{1,j} = \sqrt{2S}b^\dagger_{1,j}, \quad \tilde{S}^{B,x}_{1,j} + i\tilde{S}^{B,y}_{1,j} = \sqrt{2S}b_{1,j}$$

$$\tilde{S}^{A,z}_{2,j} = S - a^\dagger_{2,j}a_{2,j}, \quad \tilde{S}^{A,x}_{2,j} - i\tilde{S}^{A,y}_{2,j} = \sqrt{2S}a^\dagger_{2,j}, \quad \tilde{S}^{A,x}_{2,j} + i\tilde{S}^{A,y}_{2,j} = \sqrt{2S}a_{2,j}$$

$$\tilde{S}^{B,z}_{2,j} = S - b^\dagger_{2,j}b_{2,j}, \quad \tilde{S}^{B,x}_{2,j} - i\tilde{S}^{B,y}_{2,j} = \sqrt{2S}b^\dagger_{2,j}, \quad \tilde{S}^{B,x}_{2,j} + i\tilde{S}^{B,y}_{2,j} = \sqrt{2S}b_{2,j}$$

where $\left(\tilde{S}^{A,x}_{\mathrm{j}}, \tilde{S}^{A,y}_{\mathrm{j}}, \tilde{S}^{A,z}_{\mathrm{j}}\right)$ and $\left(\tilde{S}^{B,x}_{\mathrm{j}}, \tilde{S}^{B,y}_{\mathrm{j}}, \tilde{S}^{B,z}_{\mathrm{j}}\right)$ are the spin operators in a rotated frame:

$$\begin{pmatrix} \tilde{S}^{\alpha,x}_{1,j} \\ \tilde{S}^{\alpha,y}_{1,j} \\ \tilde{S}^{\alpha,z}_{1,j} \end{pmatrix} = \begin{pmatrix} 1 & 0 & 0 \\ 0 & cos\psi & -sin\psi \\ 0 & sin\psi & cos\psi \end{pmatrix} \begin{pmatrix} \mathrm{S}^{\alpha,\mathrm{x}}_{1,\mathrm{j}} \\ \mathrm{S}^{\alpha,\mathrm{y}}_{1,\mathrm{j}} \\ \mathrm{S}^{\alpha,\mathrm{z}}_{1,\mathrm{j}} \end{pmatrix}$$

$$\begin{pmatrix} \tilde{S}^{\alpha,x}_{2,j} \\ \tilde{S}^{\alpha,y}_{2,j} \\ \tilde{S}^{\alpha,z}_{2,j} \end{pmatrix} = \begin{pmatrix} 1 & 0 & 0 \\ 0 & -cos\psi & -sin\psi \\ 0 & sin\psi & -cos\psi \end{pmatrix} \begin{pmatrix} \mathrm{S}^{\alpha,\mathrm{x}}_{2,\mathrm{j}} \\ \mathrm{S}^{\alpha,\mathrm{y}}_{2,\mathrm{j}} \\ \mathrm{S}^{\alpha,\mathrm{z}}_{2,\mathrm{j}} \end{pmatrix}$$

Here $\alpha = A, B$, a and b are Holstein-Primakoff boson fields for A-sublattice Cr spin and B-sublattice Cr spin, that represent fluctuations around the classical magnetic order. Around those ψ that minimize the classical magnetic energy, the Hamiltonian is stable against such small fluctuations:

$$H \equiv E_{classical} + H_{sw} + \mathcal{O}(a^3, b^3)$$

That says, a spin-wave Hamiltonian H_{sw} is given by a quadratic form in the boson fields. We use equation S8 to replace h by J and D, and transform the spin-wave Hamiltonian into the momentum space:

$$H_{magnon} = \Psi^{+}(\mathbf{k})\begin{bmatrix} M_0 & f(k) & N_2 & 0 & N_0 & 0 & N_1 & 0 \\ f^*(k) & M_0 & 0 & N_2 & 0 & N_0 & 0 & N_1 \\ N_2 & 0 & M_0 & f(k) & N_1 & 0 & N_0 & 0 \\ 0 & N_2 & f^*(k) & M_0 & 0 & N_1 & 0 & N_0 \\ N_0 & 0 & N_1 & 0 & M_0 & f(k) & N_2 & 0 \\ 0 & N_0 & 0 & N_1 & f^*(k) & M_0 & 0 & N_2 \\ N_1 & 0 & N_0 & 0 & N_2 & 0 & M_0 & f(k) \\ 0 & N_1 & 0 & N_0 & 0 & N_2 & f^*(k) & M_0 \end{bmatrix}\Psi(\mathbf{k})$$

with $\Psi(\mathbf{k}) = \left(a_1(\mathbf{k}), b_1(\mathbf{k}), a_2(\mathbf{k}), b_2(\mathbf{k}), a_1^{+}(-\mathbf{k}), b_1^{+}(-\mathbf{k}), a_2^{+}(-\mathbf{k}), b_2^{+}(-\mathbf{k})\right)^T$, $M_0 = \frac{-J_1S-J_2S-2J_3S}{2} + DScos^2\psi - \frac{DS}{2}\sin^2\psi + \frac{h}{2}sin\psi + \frac{J_cS}{2}cos2\psi$, $N_0 = \frac{DS}{2}\sin^2\psi$, $N_1 = \frac{J_cS}{4}(1+cos2\psi)$, $N_2 = \frac{J_cS}{4}(1-cos2\psi)$, $|f(\mathbf{k})|$ and $\varphi_{\mathbf{k}}$ are the modulus and phase of $f(\mathbf{k}) = \frac{S}{2}\sum_{m=1,2,3,4} J_{a_m} e^{i\mathbf{k}\mathbf{a_m}} \equiv |f(k)|e^{i\varphi_k}$ respectively.

Under

$$\Psi(\mathbf{k}) = \sigma_0 \otimes \begin{pmatrix} 1 & -1 \\ 1 & 1 \end{pmatrix} \otimes \frac{1}{2}\begin{pmatrix} e^{i\varphi_k} & -e^{i\varphi_k} \\ 1 & 1 \end{pmatrix}\Psi'(\mathbf{k})$$

$$\begin{pmatrix} \Psi_i'(\mathbf{k}) \\ \Psi_i'^{+}(-\mathbf{k}) \end{pmatrix} = \begin{pmatrix} \cosh\frac{\delta_i}{2} & -\sinh\frac{\delta_i}{2} \\ -\sinh\frac{\delta_i}{2} & \cosh\frac{\delta_i}{2} \end{pmatrix}\begin{pmatrix} \gamma_i(\mathbf{k}) \\ \gamma_i^{\dagger}(-\mathbf{k}) \end{pmatrix}, i = 1,2,3,4$$

The spin-wave Hamiltonian is diagonalized as,

$$H_{magnon} = \sum_k (\hbar\omega_1(\mathbf{k})\gamma_1^{\dagger}(\mathbf{k})\gamma_1(\mathbf{k}) + \hbar\omega_2(\mathbf{k})\gamma_2^{\dagger}(\mathbf{k})\gamma_2(\mathbf{k}) + \hbar\omega_3(\mathbf{k})\gamma_3^{\dagger}(\mathbf{k})\gamma_3(\mathbf{k}) + \hbar\omega_4(\mathbf{k})\gamma_4^{\dagger}(\mathbf{k})\gamma_4(\mathbf{k}))$$

Here,

$$\cosh\delta_1 = \frac{M_0-N_2-|f(\mathbf{k})|}{\hbar\omega_1(\mathbf{k})} , \quad \sinh\delta_1 = -\frac{N_0-N_1}{\hbar\omega_1(\mathbf{k})}$$

$$\cosh\delta_2 = \frac{M_0+N_2-|f(\mathbf{k})|}{\hbar\omega_2(\mathbf{k})} , \quad \sinh\delta_2 = -\frac{N_0+N_1}{\hbar\omega_2(\mathbf{k})}$$

$$\cosh\delta_3 = \frac{M_0-N_2+|f(\mathbf{k})|}{\hbar\omega_3(\mathbf{k})} , \quad \sinh\delta_3 = -\frac{N_0-N_1}{\hbar\omega_3(\mathbf{k})}$$

$$\cosh\delta_4 = \frac{M_0+N_2+|f(\mathbf{k})|}{\hbar\omega_4(\mathbf{k})} , \quad \sinh\delta_4 = -\frac{N_0+N_1}{\hbar\omega_4(\mathbf{k})}$$

$$E_1 = \hbar\omega_1(\mathbf{k}) = \sqrt{(|M_0 - N_2| - |f(\mathbf{k})|)^2 - (N_0 - N_1)^2}$$

$$E_2 = \hbar\omega_2(\mathbf{k}) = \sqrt{(|M_0 + N_2| - |f(\mathbf{k})|)^2 - (N_0 + N_1)^2}$$

$$E_3 = \hbar\omega_3(\mathbf{k}) = \sqrt{(|M_0 - N_2| + |f(\mathbf{k})|)^2 - (N_0 - N_1)^2}$$

$$E_4 = \hbar\omega_4(\mathbf{k}) = \sqrt{(|M_0 + N_2| + |f(\mathbf{k})|)^2 - (N_0 + N_1)^2} \quad \text{(S9)}$$

Because the weak interlayer magnetic exchange coupling, N_0, N_1, N_2 is much smaller than other parameters. Thus, band E_1 and $E_2$is close to degeneracy, and band E_3 and E_4 is close to degeneracy. The lowest E_1 spin-wave energy momentum dispersions along high symmetric momentum line are plotted in Fig. S8.2. A highly anisotropic dispersion is resulted by the anisotropy of magnetic exchange coupling. It is obvious that the band dispersion along k_y (<010>) direction (with stronger magnetic coupling) has a larger group velocity $\mathbf{v} = \frac{\partial \varepsilon}{\partial k}$, which will give larger spin current and corresponding SHM signal.

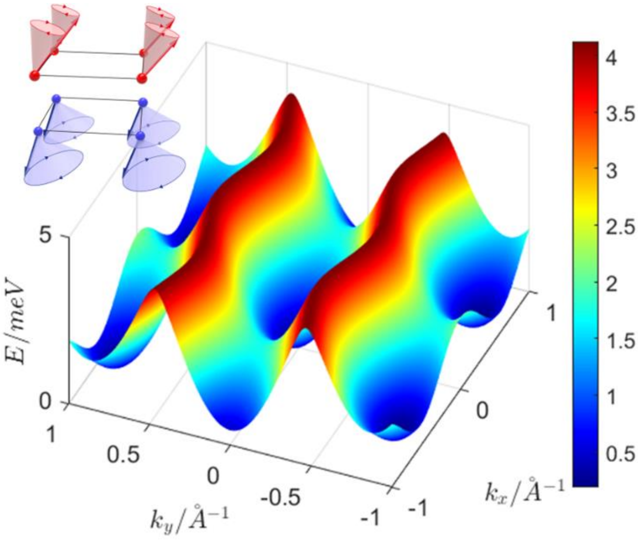

Fig. S8.2. Spin model and spin wave modes of $CrPS_4$ under an in-plane magnetic field. Anisotropic E_1 magnon band structure of bilayer $CrPS_4$ when the in-plane anisotropic nearest neighbor coupling J_1=-2.96meV, J_2=-2.09meV, J_3=-0.51meV, interlayer coupling J_c=0.16meV, the magnetic anisotropy D_z = 0.0058meV and an in-plane magnetic field of 4T along the *x* direction.

S9. Modeling spin Seebeck effect in $CrPS_4$

Based on a semi-classical Boltzmann transport theory of magnon in anti-ferromagnet[7], we will give an expression for spin Seebeck coefficient of $CrPS_4$ at finite temperature and finite magnetic field in this section.

Here we first consider the experimental configuration for Device-S, where the magnetic field is along *y* axis. The spin density along the field direction is given by magnon creation and annihilation operators:

$$\sum_{\mathbf{j}}\left(S_{\mathbf{1,j}}^{A,y}+S_{\mathbf{1,j}}^{B,y}+S_{\mathbf{2,j}}^{A,y}+S_{\mathbf{2,j}}^{B,y}\right)$$

$$=\sum_{\mathbf{j}}\Big(\cos\psi\,\tilde{S}_{\mathbf{1,j}}^{A,y}+\sin\psi\,\tilde{S}_{\mathbf{1,j}}^{A,z}+\cos\psi\,\tilde{S}_{\mathbf{1,j}}^{B,y}+\sin\psi\,\tilde{S}_{\mathbf{1,j}}^{B,z}+\cos\psi\,\tilde{S}_{\mathbf{2,j}}^{A,y}$$

$$-\sin\psi\,\tilde{S}_{\mathbf{2,j}}^{A,z}+\cos\psi\,\tilde{S}_{\mathbf{2,j}}^{B,y}-\sin\psi\,\tilde{S}_{\mathbf{2,j}}^{B,z}\Big)$$

$$=\sin\psi\sum_{\mathbf{j}}\left(\tilde{S}_{\mathbf{1,j}}^{A,z}+\tilde{S}_{\mathbf{1,j}}^{B,z}\right)+\cos\psi\sum_{\mathbf{j}}\left(\tilde{S}_{\mathbf{1,j}}^{A,y}+\tilde{S}_{\mathbf{1,j}}^{B,y}\right)-\sin\psi\sum_{\mathbf{j}}\Big(\tilde{S}_{\mathbf{2,j}}^{A,z}+$$

$$\tilde{S}_{\mathbf{2,j}}^{B,z}\Big)+\cos\psi\sum_{\mathbf{j}}\left(\tilde{S}_{\mathbf{2,j}}^{A,y}+\tilde{S}_{\mathbf{2,j}}^{B,y}\right)$$

$$=\sin\psi\sum_{\mathbf{j}}\left(2S-a_{\mathbf{1,j}}^{\dagger}a_{\mathbf{1,j}}-b_{\mathbf{1,j}}^{\dagger}b_{\mathbf{1,j}}-a_{\mathbf{2,j}}^{\dagger}a_{\mathbf{2,j}}-b_{\mathbf{2,j}}^{\dagger}b_{\mathbf{2,j}}\right)+$$

$$\cos\psi\sum_{\mathbf{j}}\sqrt{\frac{S}{2}}\left(a_{\mathbf{1,j}}^{\dagger}+a_{\mathbf{1,j}}+b_{\mathbf{1,j}}^{\dagger}+b_{\mathbf{1,j}}+a_{\mathbf{2,j}}^{\dagger}+a_{\mathbf{2,j}}+b_{\mathbf{2,j}}^{\dagger}+b_{\mathbf{2,j}}\right).\quad\text{(S10)}$$

The second terms in the last line are linear in the magnon creation or annihilation operators, so that they are time-dependent with a factor of $e^{\pm i\,\hbar\omega_{1,2,3,4}(\mathbf{k})t+i\phi}$. Within the experimental measurement resolution, these contributions must be averaged to the zero. Besides, the initial phase ϕ in the time-dependent factor are equally distributed with $[0,2\pi]$, because the magnon are supposed to be thermally activated under the injector. After being averaged over the initial ϕ, the contributions must be zero too. Thus, we consider the contribution of the first term in equation S10 and obtain the average spin projection along the magnetic field as:

$$\sum_j \langle S_{1,j}^{A,y} + S_{1,j}^{B,y} + S_{2,j}^{A,y} + S_{2,j}^{B,y} \rangle$$

$$= -sin\psi \sum_k \langle \cosh\delta_1 \gamma_1^\dagger(\mathbf{k})\gamma_1(\mathbf{k}) + \cosh\delta_2 \gamma_2^\dagger(\mathbf{k})\gamma_2(\mathbf{k})$$

$$+ \cosh\delta_3 \gamma_3^\dagger(\mathbf{k})\gamma_3(\mathbf{k}) + \cosh\delta_4 \gamma_4^\dagger(\mathbf{k})\gamma_4(\mathbf{k}) \rangle$$

Note that the same signs in angular momenta of the four magnon modes, $\cosh\delta_1$, $\cosh\delta_2$, $\cosh\delta_3$, $\cosh\delta_4 > 0$. Since the two magnon modes have different group velocity $\mathbf{v_j}(\mathbf{k}) = \nabla_{\mathbf{k}}\omega_j(\mathbf{k})$ $(j = 1,2,3,4)$, the spin current density operator J_m is:

$$J_S^y = -\frac{\hbar}{(2\pi)^2} \sin\psi \int \mathrm{dk_x} dk_y \sum_{i=1}^{4} \mathbf{v_i}(\mathbf{k}) cosh\delta_i [n_i(\mathbf{k}) - n_i^0(\mathbf{k})]$$

$$n_i(\mathbf{k}) - n_i^0(\mathbf{k}) = -\tau_i(\mathbf{k})\mathbf{v_i}(\mathbf{k}) \cdot \nabla n_i^0(\mathbf{k})$$

For Device-W, a similar J_S^x could be obtained. We follow the same argument as Ref.[7], to obtain the spin Seebeck coefficient:

$$\boldsymbol{J}_m = \boldsymbol{S} \cdot \nabla T$$

$$\boldsymbol{S}(T) = \frac{\hbar}{(2\pi)^2 k_B T^2} sin\psi \int_{BZ} dk_x dk_y \sum_{i=1}^{4} \boldsymbol{v}_i(\boldsymbol{k}) cosh\delta_i \boldsymbol{v}_i(\boldsymbol{k}) \frac{e^{\frac{\hbar\omega_i}{k_B T}} \hbar\omega_i \tau_i(\boldsymbol{k})}{\left(e^{\frac{\hbar\omega_i}{k_B T}} - 1\right)^2} \quad \text{(S11)}$$

where $\eta_{i,k} = 1/\tau_{i,k}$ is the magnon relaxation time and $n_i(k) = \langle \gamma_i^\dagger(k)\gamma_\mathrm{i}(k) \rangle$ is the magnon density for the ith magnon branch and at magnon momentum $\boldsymbol{k}$. $\mathbf{v}_i(\boldsymbol{k})\mathbf{v}_i(\boldsymbol{k})$ is generally in the tensor-form and so is the Seebeck coefficient **S**. The dispersion relation of the four magnon branches is given in equation S9.

Thus, we are ready to simulate $V_{2\omega,0}^S$ and $V_{2\omega,0}^W$ as[7, 8, 9]:

$$V_{2\omega,0}^{S,W} = C^{S,W} * [\,\hat{\boldsymbol{n}} \cdot \boldsymbol{S}(T) \cdot \nabla T]_{2\omega}$$

$$= C^{S,W} * \left[\, S_n\left(2\mathrm{K} + c_1^{S,W} I_{in}^2 + c_2^{S,W} I_{gate}^2\right) * \left(c_1^{S,W} I_{in}^2 + c_2^{S,W} I_{gate}^2\right)\right]_{2\omega} \quad \text{(S12)}$$

where, as stated in the main text, $\hat{\boldsymbol{n}} = \hat{\boldsymbol{x}}, \hat{\boldsymbol{y}}$ are unitary vectors corresponds magnon

propagation directions in Device-W and Device-S, $C^{S,W}$ is an overall constant, $c_1^{S,W}$ and $c_2^{S,W}$ are heating efficiency of the injector and gate along the two directions, respectively, and $[\ldots]_{2\omega}$ means taking the second harmonic component.

S10. Ignorable out-of-plane spin Seebeck coefficient $\boldsymbol{S_z(T)}$

We extend our model in Supplementary S8 and S9 to three-dimension and calculate the out-of-plane spin Seebeck coefficient $\boldsymbol{S_z}(T)$. Magnetism for the bulk $CrPS_4$ is described by the following Hamiltonian:

$$H = \sum_{\boldsymbol{j}} \sum_{m=1,2,3,4} J_{\boldsymbol{a}_m} \boldsymbol{S}_{\boldsymbol{j}}^A \cdot \boldsymbol{S}_{\boldsymbol{j}+\boldsymbol{a}_m}^B - D \sum_{\boldsymbol{j}} \left[\left(S_{\boldsymbol{j}}^{A,z}\right)^2 + \left(S_{\boldsymbol{j}}^{B,z}\right)^2 \right] - h \sum_{\boldsymbol{j}} \left[S_{\boldsymbol{j}}^{A,y} + S_{\boldsymbol{j}}^{B,y} \right]$$

$$+ \sum_{\boldsymbol{j}} J_c \left(\boldsymbol{S}_{\boldsymbol{j}}^A \cdot \boldsymbol{S}_{\boldsymbol{j}+\boldsymbol{a}_5}^A + \boldsymbol{S}_{\boldsymbol{j}}^B \cdot \boldsymbol{S}_{\boldsymbol{j}+\boldsymbol{a}_5}^B \right)$$

Here D is the easy-axis single-ion anisotropy and J is the magnetic exchange coupling with $J_{a_1} = J_1$, $J_{a_2} = J_2$, $J_{a_3} = J_3$, $J_{a_4} = J_3$. $\boldsymbol{j}$ denotes a monoclinic-lattice A-sublattice site, and $\boldsymbol{a}_m$ $(m = 1,2,3,4,5)$ connects a A-sublattice site and its neighboring four B-sublattice sites with $\boldsymbol{a}_1 = 0$, $\boldsymbol{a}_2 = \boldsymbol{e}_2, \boldsymbol{a}_3 = \boldsymbol{e}_1, \boldsymbol{a}_4 = \boldsymbol{e}_2 - \boldsymbol{e}_1$. $\boldsymbol{a}_5 = \boldsymbol{e}_3$ is the normal vector connected two adjacent layers. $\boldsymbol{S}_j^{\mathrm{A}} \equiv \left(S_{\boldsymbol{j}}^{A,x}, S_{\boldsymbol{j}}^{A,y}, S_{\boldsymbol{j}}^{A,z}\right)$ is a localized spin of Cr atom (S=3/2) in the A-sublattice site ($\boldsymbol{j}$) and $\boldsymbol{S}_{\boldsymbol{j}+\boldsymbol{a}_m}^B$ is a localized Cr spin at the B-sublattice site$(\boldsymbol{j} + \boldsymbol{a}_m)$.

Under the in-plane field h, the antiferromagnetically aligned spins will be canted linearly towards the field direction:

$$\boldsymbol{S}_{\boldsymbol{j}}^A = \boldsymbol{S}_{\boldsymbol{j}}^B = S(0, sin\psi, \cos\psi)|_{\boldsymbol{j}_z = 2n\mathbf{e}_3}$$

$$\boldsymbol{S}_{\boldsymbol{j}}^A = \boldsymbol{S}_{\boldsymbol{j}}^B = S(0, sin\psi, -\cos\psi)|_{\boldsymbol{j}_z = (2n+1)\mathbf{e}_3}$$

with *2n* denotes even layers and *2n+1* denotes odd layers. A canting angle is determined classically as a minimum of a classical magnetic energy:

$$E_{classical} = N(2J_c S^2 (\sin^2\psi - \cos^2\psi) - 2DS^2 \cos^2\psi - 2hS\sin\psi),$$

where N is a number of the A-sublattice sites. The minimum energy is given by:

$$\sin\psi = \frac{h}{2(2J_c + D)S}$$

Magnetic collective excitations around the classical magnetic order are described by the Holstein-Primakoff bosons:

$$\tilde{S}_j^{A,z} = S - a_j^\dagger a_j, \qquad \tilde{S}_j^{A,x} - i\tilde{S}_j^{A,y} = \sqrt{2S}a_j^\dagger, \qquad \tilde{S}_j^{A,x} + i\tilde{S}_j^{A,y} = \sqrt{2S}a_j$$

$$\tilde{S}_j^{B,z} = S - b_j^\dagger b_j, \qquad \tilde{S}_j^{B,x} - i\tilde{S}_j^{B,y} = \sqrt{2S}b_j^\dagger, \qquad \tilde{S}_j^{B,x} + i\tilde{S}_j^{B,y} = \sqrt{2S}b_j$$

where $\left(\tilde{S}_j^{A,x}, \tilde{S}_j^{A,y}, \tilde{S}_j^{A,z}\right)$ and $\left(\tilde{S}_j^{B,x}, \tilde{S}_j^{B,y}, \tilde{S}_j^{B,z}\right)$ are the spin operators in a rotated frame:

$$\begin{pmatrix} \tilde{S}_j^{\alpha,x} \\ \tilde{S}_j^{\alpha,y} \\ \tilde{S}_j^{\alpha,z} \end{pmatrix} = \begin{pmatrix} 1 & 0 & 0 \\ 0 & cos\psi & -sin\psi \\ 0 & sin\psi & cos\psi \end{pmatrix} \begin{pmatrix} \mathrm{S}_\mathrm{j}^{\alpha,\mathrm{x}} \\ \mathrm{S}_\mathrm{j}^{\alpha,\mathrm{y}} \\ \mathrm{S}_\mathrm{j}^{\alpha,\mathrm{z}} \end{pmatrix} |_{j_z = 2n\mathbf{e}_3}$$

$$\begin{pmatrix} \tilde{S}_j^{\alpha,x} \\ \tilde{S}_j^{\alpha,y} \\ \tilde{S}_j^{\alpha,z} \end{pmatrix} = \begin{pmatrix} 1 & 0 & 0 \\ 0 & -cos\psi & -sin\psi \\ 0 & sin\psi & -cos\psi \end{pmatrix} \begin{pmatrix} \mathrm{S}_\mathrm{j}^{\alpha,\mathrm{x}} \\ \mathrm{S}_\mathrm{j}^{\alpha,\mathrm{y}} \\ \mathrm{S}_\mathrm{j}^{\alpha,\mathrm{z}} \end{pmatrix} |_{j_z = (2n+1)\mathbf{e}_3}$$

Here $\alpha = A, B$, a and b are Holstein-Primakoff boson fields for A-sublattice Cr spins and B-sublattice Cr spins, that represent fluctuations around the classical magnetic order. Around those ψ that minimize the classical magnetic energy, the Hamiltonian is stable against such small fluctuations:

$$H \equiv E_{classical} + H_{sw} + O(a^3, b^3)$$

Thus, the spin-wave Hamiltonian transformed into the momentum space (here **k** is a three-dimensional wave vector) is:

$$H_{magnon} = \sum_{\boldsymbol{k}} \Psi^+(\mathbf{k}) \begin{bmatrix} M_0 & f(\mathbf{k}) & N_0 & 0 \\ f^*(\mathbf{k}) & M_0 & 0 & N_0 \\ N_0 & 0 & M_0 & f(\mathbf{k}) \\ 0 & N_0 & f^*(\mathbf{k}) & M_0 \end{bmatrix} \Psi(\mathbf{k})$$

with $\Psi(\mathbf{k}) = \left(a(\mathbf{k}), b(\mathbf{k}), a^+(-\mathbf{k}), b^+(-\mathbf{k})\right)^T$, $M_0 = \frac{-J_1S - J_2S - 2J_3S}{2} + DScos^2\psi - \frac{DS}{2}\sin^2\psi + \frac{h}{2}sin\psi + J_cScos2\psi + \frac{J_cS}{2}(1 - cos2\psi)\cos(\mathbf{k}\cdot\mathbf{a}_5)$, $N_0 = \frac{DS}{2}\sin^2\psi + \frac{J_cS}{2}(1 + cos2\psi)\cos(\mathbf{k}\cdot\mathbf{a}_5)$, $|f(\mathbf{k})|$ and $\varphi_{\boldsymbol{k}}$ are the modulus and phase of $f(\mathbf{k}) =$

$\frac{S}{2}\sum_{m=1,2,3,4} J_{a_m} e^{i\boldsymbol{k}\cdot\boldsymbol{a}_m} \equiv |f(\mathbf{k})|e^{i\varphi_{\boldsymbol{k}}}$ respectively.

Under

$$\begin{pmatrix} a(\mathbf{k}) \\ b(\mathbf{k}) \\ a^{+}(-\mathbf{k}) \\ b^{+}(-\mathbf{k}) \end{pmatrix} = \sigma_0 \otimes \frac{1}{\sqrt{2}} \begin{pmatrix} e^{i\frac{\varphi_{\boldsymbol{k}}}{2}} & 0 \\ 0 & e^{-i\frac{\varphi_{\boldsymbol{k}}}{2}} \end{pmatrix} \begin{pmatrix} 1 & -1 \\ 1 & 1 \end{pmatrix} \begin{pmatrix} \alpha_{\boldsymbol{k}} \\ \beta_{\boldsymbol{k}} \\ \alpha_{-\boldsymbol{k}}^{\dagger} \\ \beta_{-\boldsymbol{k}}^{\dagger} \end{pmatrix}$$

$$\begin{pmatrix} \alpha_{\boldsymbol{k}} \\ \alpha_{-\boldsymbol{k}}^{\dagger} \end{pmatrix} = \begin{pmatrix} \cosh\frac{\delta_1}{2} & -\sinh\frac{\delta_1}{2} \\ -\sinh\frac{\delta_1}{2} & \cosh\frac{\delta_1}{2} \end{pmatrix} \begin{pmatrix} \gamma_{1,\boldsymbol{k}} \\ \gamma_{1,-\boldsymbol{k}}^{\dagger} \end{pmatrix}, \begin{pmatrix} \beta_{\boldsymbol{k}} \\ \beta_{-\boldsymbol{k}}^{\dagger} \end{pmatrix}$$

$$= \begin{pmatrix} \cosh\frac{\delta_2}{2_2} & \sinh\frac{\delta_2}{2_2} \\ \sinh\frac{\delta_2}{2} & \cosh\frac{\delta_2}{2} \end{pmatrix} \begin{pmatrix} \gamma_{2,\boldsymbol{k}} \\ \gamma_{2,-\boldsymbol{k}}^{\dagger} \end{pmatrix}$$

The spin-wave Hamiltonian is diagonalized as,

$$H_{magnon} = \sum_{\boldsymbol{k}} (\hbar\omega_1(\mathbf{k})\gamma_1^{\dagger}(\mathbf{k})\gamma_1(\mathbf{k}) + \hbar\omega_2(\mathbf{k})\gamma_2^{\dagger}(\mathbf{k})\gamma_2(\mathbf{k}))$$

Here,

$$\cosh\delta_1 = \frac{M_0 + |f(\mathbf{k})|}{E_1}$$

$$\cosh\delta_2 = \frac{M_0 - |f(\mathbf{k})|}{E_1}$$

$$E_1(\mathbf{k}) = \hbar\omega_1(\mathbf{k}) = \sqrt{(M_0 + |f(\mathbf{k})|)^2 - N_0^2}$$

$$E_2(\mathbf{k}) = \hbar\omega_2(\mathbf{k}) = \sqrt{(M_0 - |f(\mathbf{k})|)^2 - N_0^2}.$$

The lowest E_2 spin-wave energy momentum dispersions with $k_y = 0$ or $k_x = 0$ are plotted in Fig. S10. As can be seen, the energy band is nearly dispersionless along k_z direction compared to k_x and k_y.

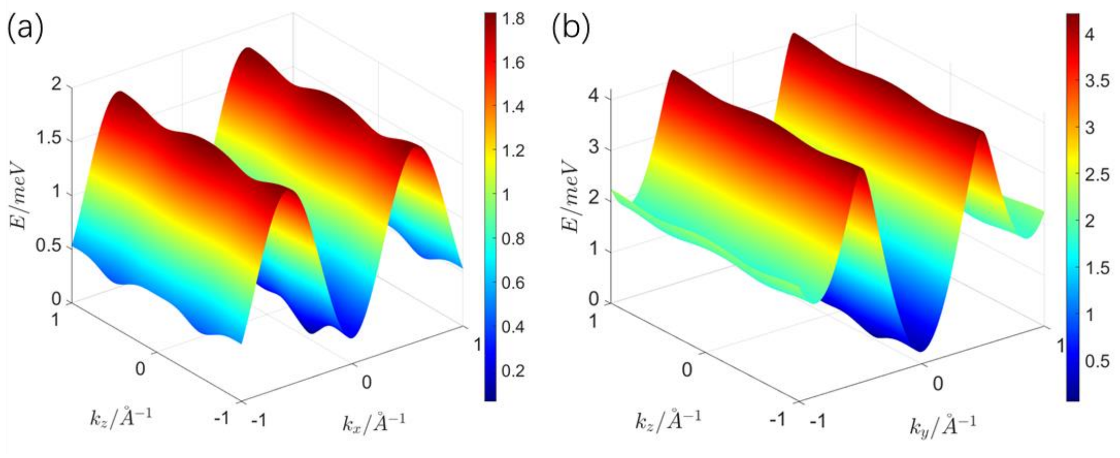

Fig. S10.1. Three-dimensional spin model and spin wave modes of $CrPS_4$ under an in-plane magnetic field. (a) The lowest E_2 spin-wave energy momentum dispersions with $k_y = 0$. (b) The lowest E_2 spin-wave energy momentum dispersions with $k_x = 0$.

To get the expression for spin Seebeck coefficient, we first consider the experimental configuration for Device-S, where the magnetic field is along the y axis. The spin density along the field direction is given by magnon creation and annihilation operators:

$$\sum_{\boldsymbol{j}}\left(S_{\boldsymbol{j}}^{A,y}+S_{\boldsymbol{j}}^{B,y}\right)=sin\psi\sum_{\boldsymbol{j}}(2S-a_{\boldsymbol{j}}^{\dagger}a_{\boldsymbol{j}}-b_{\boldsymbol{j}}^{\dagger}b_{\boldsymbol{j}})+cos\psi\sum_{\boldsymbol{j}}\sqrt{\frac{S}{2}}(a_{\boldsymbol{j}}^{\dagger}+a_{\boldsymbol{j}}+b_{\boldsymbol{j}}^{\dagger}+b_{\boldsymbol{j}})$$

We consider the contribution of the first term (the second linear term vanishes in average) and obtain the average spin projection along the magnetic field as:

$$\sum_{j}\langle S_{j}^{A,y}+S_{j}^{B,y}\rangle = -sin\psi\sum_{\boldsymbol{k}}\langle \cosh\delta_1\gamma_1^{\dagger}(\mathbf{k})\gamma_1(\mathbf{k})+\cosh\delta_2\gamma_2^{\dagger}(\mathbf{k})\gamma_2(\mathbf{k})\rangle$$

With $n_i(\mathbf{k})=\langle\gamma_i^{\dagger}(\mathbf{k})\gamma_{\mathrm{i}}(\mathbf{k})\rangle$, the spin current density operator J_m is:

$$J_S^y=-\frac{\hbar}{(2\pi)^3}\sin\psi\int \mathrm{d}k_{\mathrm{x}}dk_ydk_z\sum_{i=1}^{2}v_i(\boldsymbol{k})cosh\delta_i[n_i(\boldsymbol{k})-n_i^0(\boldsymbol{k})]$$

$$n_i(\boldsymbol{k})-n_i^0(\boldsymbol{k})=-\tau_i(\boldsymbol{k})v_i(\boldsymbol{k})\cdot\nabla n_i^0(\boldsymbol{k})$$

For Device-W, a similar J_S^x could be obtained. Based on $\boldsymbol{J}_m=\boldsymbol{S}\cdot\boldsymbol{\nabla}T$, the three-dimensional spin Seebeck coefficient reads:

$$\boldsymbol{S}(T) = \frac{\hbar}{(2\pi)^3 k_B T^2} sin\psi \int_{BZ} dk_x dk_y dk_z \sum_{i=1}^{2} \mathbf{v_i}(\mathbf{k}) cosh\delta_i \mathbf{v_i}(\mathbf{k}) \frac{e^{\frac{\hbar\omega_i}{k_B T}} \hbar\omega_i \tau_i(\mathbf{k})}{\left(e^{\frac{\hbar\omega_i}{k_B T}} - 1\right)^2}$$

Figure S10.2 shows the calculated spin Seebeck coefficient $S_n(T)$. The out-of-plane spin Seebeck coefficient $S_z(T)$ is quite small compared with the in-plane coefficients $S_x(T)$ and $S_y(T)$ at T = 2K and above. This 2D nature of the magnon transport in $CrPS_4$ is warranted by the weak interlayer exchange interaction.

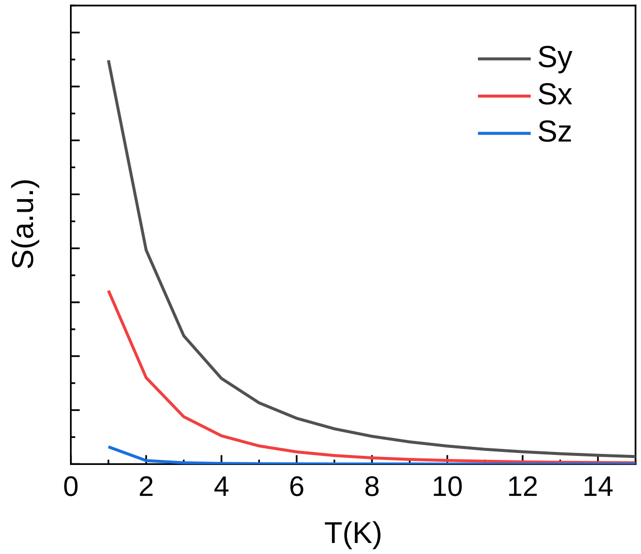

Fig. S10.2. The simulated spin Seebeck coefficient S_n with a three-dimensional model.

Since both of the out-of-plane temperature gradient under the detector (see Supplementary S11) and the spin Seebeck coefficient $S_z(T)$ are small, the two-dimensional spin transport model could capture the majority of the physics and thus the Equation 2 in the main text is a good approximation at the moment. It is indeed interesting to provide a more comprehensive theory to the experimental result we obtained in this manuscript, and we hope that our experimental work could stimulate further discussions and theoretical works in the field.

S11. Finite element analysis of the temperature and spin chemical potential distribution in $CrPS_4$ device

To clarify how the heat flows in the sample and how it affects the distribution of the temperature gradience in the sample in the in-plane and out-of-plane directions, we perform additional finite element analyses (*Phys. Rev. B* 96, 104441 (2017)) of the temperature distribution and magnon chemical potential distribution for $CrPS_4$ magnon valve devices with crystal thickness of 30nm.

The linear response relation of heat and spin transport in the bulk of a magnetic insulator reads:

$$\begin{pmatrix} \frac{2e}{\hbar}\boldsymbol{j}_m \\ \boldsymbol{j}_Q \end{pmatrix} = -\begin{pmatrix} \sigma_m & \boldsymbol{S}/T \\ \boldsymbol{S} & \kappa \end{pmatrix}\begin{pmatrix} \boldsymbol{\nabla}\mu_m \\ \boldsymbol{\nabla}T \end{pmatrix}$$

where $\boldsymbol{j}_m$ is the magnon spin current, $\boldsymbol{j}_Q$ the total heat current, μ_m the magnon chemical potential, T the temperature, σ_m the magnon spin conductivity, κ the total heat conductivity and $\boldsymbol{S}$ the spin Seebeck coefficient. Combined with $\boldsymbol{\nabla}\cdot\boldsymbol{j}_Q = \frac{j_c^2}{\sigma_{Pt}}$, and $\boldsymbol{\nabla}\cdot\boldsymbol{j}_m = -\frac{\hbar\sigma_m}{2e\lambda_m^2}\mu_m$, the diffusion equations for spin and heat read:

$$\boldsymbol{S}\cdot\boldsymbol{\nabla}^2\mu_m + \kappa\cdot\boldsymbol{\nabla}^2 T = -\frac{j_c^2}{\sigma_{Pt}}$$

$$\sigma_m\cdot\boldsymbol{\nabla}^2\mu_m + \boldsymbol{S}\cdot\boldsymbol{\nabla}\left(\frac{\boldsymbol{\nabla}T}{T}\right) = \frac{\sigma_m\cdot\mu_m}{\lambda_m^2}$$

where j_c is the charge current density in the injector Pt electrode, σ_{Pt} is the electrical conductivity of the Pt electrode and λ_m the magnon spin diffusion length.

Since $CrPS_4$ is not as widely studied as YIG, some parameters are unavailable, let alone considering the anisotropy. For the parameters related to temperature gradient distribution analysis, there is no thermal conductivity data found for $CrPS_4$ in the literature. Here we use the in-plane thermal conductivity 6.3[W/(m*K)] for $MnPS_3$ as an rough estimate of the thermal conductivity for $CrPS_4$ along the <010> direction, and the through-plane thermal conductivity 1.1[W/(m*K)] for $MnPS_3$ as the through-plane thermal conductivity for $CrPS_4$. Based on our fitting parameters $c_1^S =$

$1.7 \times 10^{-4}\mathrm{K}/\mu\mathrm{A}^2$, $c_2^S = 1.2 \times 10^{-4}\mathrm{K}/\mu\mathrm{A}^2$ for Device-S (magnon transports along the <010> direction) and $c_1^W = 4.9 \times 10^{-4}\mathrm{K}/\mu\mathrm{A}^2$, $c_2^W = 3.8 \times 10^{-4}\mathrm{K}/\mu\mathrm{A}^2$ for Device-W (magnon transports along the <100> direction), an average $\boldsymbol{\nabla} T^W/\boldsymbol{\nabla} T^S$of 3 times is obtained. Since the heating power is fixed in our experiment, the <100> thermal conductivity for $CrPS_4$ is estimated to be 3 times smaller than the <010> thermal conductivity, which is 2.1[W/(m*K)].

For the parameters related to spin chemical potential distribution analysis, the ratio for the spin Seebeck coefficient components $S_y{:}S_x{:}S_z = 1{:}\,0.404{:}\,0.017$ are obtained from our model (details in our reply to reviewer's comment #2 and in Fig. R3 below). Here we use the spin Seebeck coefficient 500[A/m] for YIG as S_y for $CrPS_4$, then applying the ratio above, one can obtain $S_x = 202[\mathrm{A/m}]$ and $S_z = 8.5[\mathrm{A/m}]$. For the magnon spin conductivity $\boldsymbol{\sigma_m}$, since the calculation procedure of $\boldsymbol{\sigma_m}$ is similar to the calculation for $\boldsymbol{S}$ in our model used in Supplementary S10, would simply provide the resulting ratio in the following discussion. By adding the chemical potential μ_m generated by SSE caused magnons accumulation under the injector $n_i^0(k) = \frac{1}{e^{\frac{\hbar\omega_i - \mathrm{e}\mu_m}{k_B T}} - 1}$ in our model, the spin current density reads:

$$\boldsymbol{J}_m = \boldsymbol{S} \cdot \boldsymbol{\nabla} T + \frac{\hbar}{2\mathrm{e}} \boldsymbol{\sigma_m} \cdot \boldsymbol{\nabla} \mu_m$$

The resulted magnon spin conductivity is:

$$\boldsymbol{\sigma_m}(T) = \frac{2\mathrm{e}^2}{(2\pi)^3 k_B T} sin\psi \int_{BZ} dk_x dk_y dk_z \sum_{i=1}^{2} \boldsymbol{v}_i(\boldsymbol{k}) cosh\delta_i \boldsymbol{v}_i(\boldsymbol{k}) \frac{e^{\frac{\hbar\omega_i}{k_B T}} \tau_i(\boldsymbol{k})}{\left(e^{\frac{\hbar\omega_i}{k_B T}} - 1\right)^2}$$

Thus we could get the ratio for the magnon spin conductivity components $\sigma_{my}{:}\sigma_{mx}{:}\sigma_{mz} = 1{:}\,0.419{:}\,0.037$. Here we use the magnon spin conductivity 9000[S/m] for YIG as σ_{my} for $CrPS_4$, then $\sigma_{mx} = 3771[\mathrm{S/m}]$ and $\sigma_{mz} = 333[\mathrm{S/m}]$. As for the magnon spin diffusion length λ_m, we extracted $\lambda_{my} = 0.87\mu m$ and $\lambda_{mx} = 0.45\mu m$ from $R_{2\omega} = \frac{C_0}{\lambda_m} * \frac{\exp(d/\lambda_m)}{1-\exp(2d/\lambda_m)}$ (Nat. Phys. 11, 1022-1026 (2015)) using $R_{2\omega}^S(d = 0.75\mu m) = 0.527 nV/\mu A^2$, $R_{2\omega}^S(d = 1.5\mu m) = 0.189 nV/\mu A^2$ for Device-S and $R_{2\omega}^w(d = 0.75\mu m) = 0.352 nV/\mu A^2$, $R_{2\omega}^w(d =$

$1.5\mu m) = 0.064 nV/\mu A^2$ for Device-W. The $R_{2\omega}$ values are obtained from the liner slopes of $V_{2\omega,0}$ vs. I_{in}^2 curves for device2 shown in Fig. 2d in our main text and Fig. S3.2d in the Supplementary information. All the parameters used in the finite element analysis are listed below:

Pt	Conductivity	8.9E6[S/m]	COMSOL Material database
	thermal conductivity	71.6[W/(m*K)]	COMSOL Material database
$CrPS_4$	in-plane <010> thermal conductivity	6.3[W/(m*K)]	ACS Nano,14, 2424−2435(2020) for $MnPS_3$
	in-plane <100> thermal conductivity	2.1[W/(m*K)]	calculated
	through-plane thermal conductivity	1.1[W/(m*K)]	ACS Nano,14, 2424−2435(2020) for $MnPS_3$
	in-plane <010> spin Seebeck coefficient	500[A/m]	Phys. Rev. B 96, 104441 (2017) for YIG
	in-plane <100> spin Seebeck coefficient	202[A/m]	calculated
	through-plane spin Seebeck coefficient	8.5[A/m]	calculated
	in-plane <010> magnon spin conductivity	9000[S/m]	Phys. Rev. B 94, 180402(R) (2016) for YIG
	in-plane <100> magnon spin conductivity	3771[S/m]	calculated
	through-plane magnon spin conductivity	333[S/m]	calculated
	in-plane <010> magnon spin diffusion length	$0.87\mu m$	From experimental data
	in-plane <100> magnon spin diffusion length	$0.45\mu m$	From experimental data
SiO_2	thermal conductivity	1.38[W/(m*K)]	CRC Handbook of Chemistry and Physics (92nd ed.).p12.213
Si	thermal conductivity	130[W/(m*K)]	COMSOL Material database
$CrPS_4/SiO_2$	through-plane thermal resistance	5E-7[K*m^2/W]#	Computational Materials Science,

			142, 1–6 (2018)
Pt/$CrPS_4$	through-plane thermal resistance	1.4E-7[K*m^2/W]$^{\$}$	PHYSICAL REVIEW B 101, 205407 (2020)

Table S10. The parameters used in the finite element analysis. $^{\#}$There is no data found for $CrPS_4/SiO_2$ in the literature, we used value from through-plane thermal resistance between MoS_2/SiO_2 instead. $^{\$}$There is no data found for Pt/$CrPS_4$, we used estimated value for $CrBr_3$/Pt in the literature.

In the simulations, the sample thickness is t_{CrPS4} = 30 nm and the width of the crystal is w_{CrPS4} = 10 μm. The crystal is placed on top of a silicon substrate with a SiO_2 layer of 300 nm thick. The injector electrode has a thickness of t_{Pt} = 10 nm and a width of w_{Pt} = 250 nm. The heat current normal to the $CrPS_4$|vacuum and Pt|vacuum interfaces is set to be zero. The spin current normal to the $CrPS_4$|vacuum and $CrPS_4|SiO_2$ interfaces is also set to be zero. We have performed finite element analysis for a $MnPS_3$ device having similar configuration in *Nat. Commun.* **12**, 6279 (2021), which gives a temperature around 3K at the bottom of the Pt injector. Since here we have adopted the thermal conductivity for $MnPS_3$ as for $CrPS_4$, to simplify the simulation, the boundary condition T = 3K at the bottom of the Pt injector is used.

As shown in Figure R1, under the detector electrodes (1.5μm away from the injectors), both the in-plane temperature gradients and the out-of-plane temperature gradients are negligible for Device-S and Device-W. The large local out-of-plane temperature gradient should cause a large local SSE signal, and it is indeed detected experimentally, as shown in Fig. S6 in the Supplementary information. However, different from the local signal, the nonlocal signal is mainly caused by the thermal magnons diffusing to the detector. The resultant spin chemical potential distribution has similar behavior to YIG (*Phys. Rev. B* 96, 104441 2017) that the chemical potential changes sign because of the depletion of magnons below the injector and an accumulation of magnons at the $CrPS_4|SiO_2$ interface. The finite element analysis shows that out-of-plane temperature gradient plays a minimal role in our nonlocal

experiment.

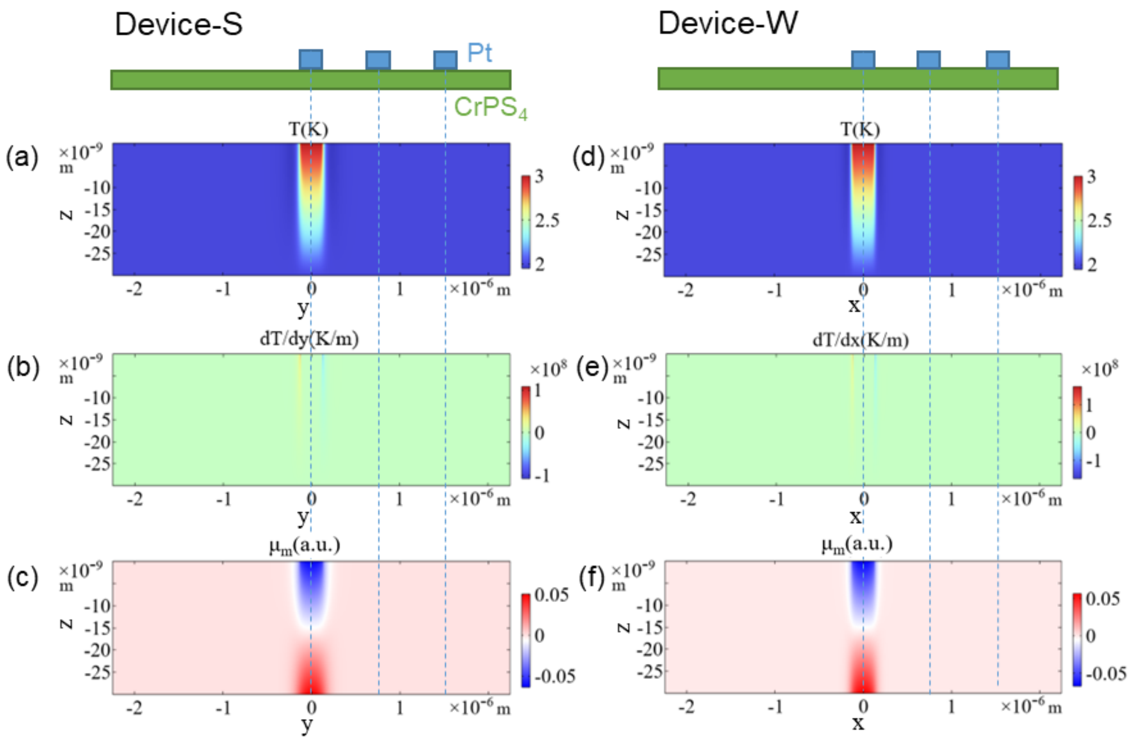

Fig. S11. Finite element analysis of the temperature and spin chemical potential distribution in $CrPS_4$ device. (a) Temperature, (b) temperature gradient and (c) spin chemical potential distribution in *yz* plane for Device-S. (d) Temperature, (e) temperature gradient and (f) spin chemical potential distribution in *xz* plane for Device-W.

S12. Simulated and experimental $V_{2\omega,0}^{S}$ and $V_{2\omega,0}^{W}$ vs. I_{gate} curves

As discussed in the previous section, the simulation of $V_{2\omega,0}^{S,W}$ can be achieved via equation (S12) with only three global parameters. Root means square deviation between the simulated curves and the experimental curves is calculated, and minimizing this deviation gives us the best values of the three global parameters: $C^S = 0.527 \times 10^{-26}\mathrm{V} \cdot \mathrm{s}/\hbar$, $c_1^S = 1.7 \times 10^{-4}\mathrm{K}/\mu\mathrm{A}^2$ and $c_2^S = 1.2 \times 10^{-4}\mathrm{K}/\mu\mathrm{A}^2$ for Device-S and $C^W = 0.484 \times 10^{-26}\mathrm{V} \cdot \mathrm{s}/\hbar$, $c_1^W = 4.9 \times 10^{-4}\mathrm{K}/\mu\mathrm{A}^2$ and $c_2^W = 3.8 \times 10^{-4}\mathrm{K}/\mu\mathrm{A}^2$ for Device-W shown in Figure 3 in the main text.

These global parameters can produce $V^S_{2\omega,0}$ and $V^W_{2\omega,0}$ curves as a function of I_{gate} under multiple values of magnetic field in Fig. S12. For the two magnon transport directions, the simulated zero crossing points I^S_0 and I^W_0 all increase monotonically with the increase of magnetic field, which is consistent with experiment. The above agreements between the simulation and the experimental data suggest that our model captures the physical trends behind the behavior of the $CrPS_4$ devices. In Supplementary Section S13 we further prove that exchange anisotropy is the key factor that produces the main features observed experimentally, while other factors, such as anisotropic thermal conductivity, anisotropic magnon scattering rate and anisotropic phonon-magnon coupling along the <100> and <010> directions in $CrPS_4$ may play a role in shaping the excellent performance of the electrically tunable anisotropy of SHM in $CrPS_4$ magnon valves.

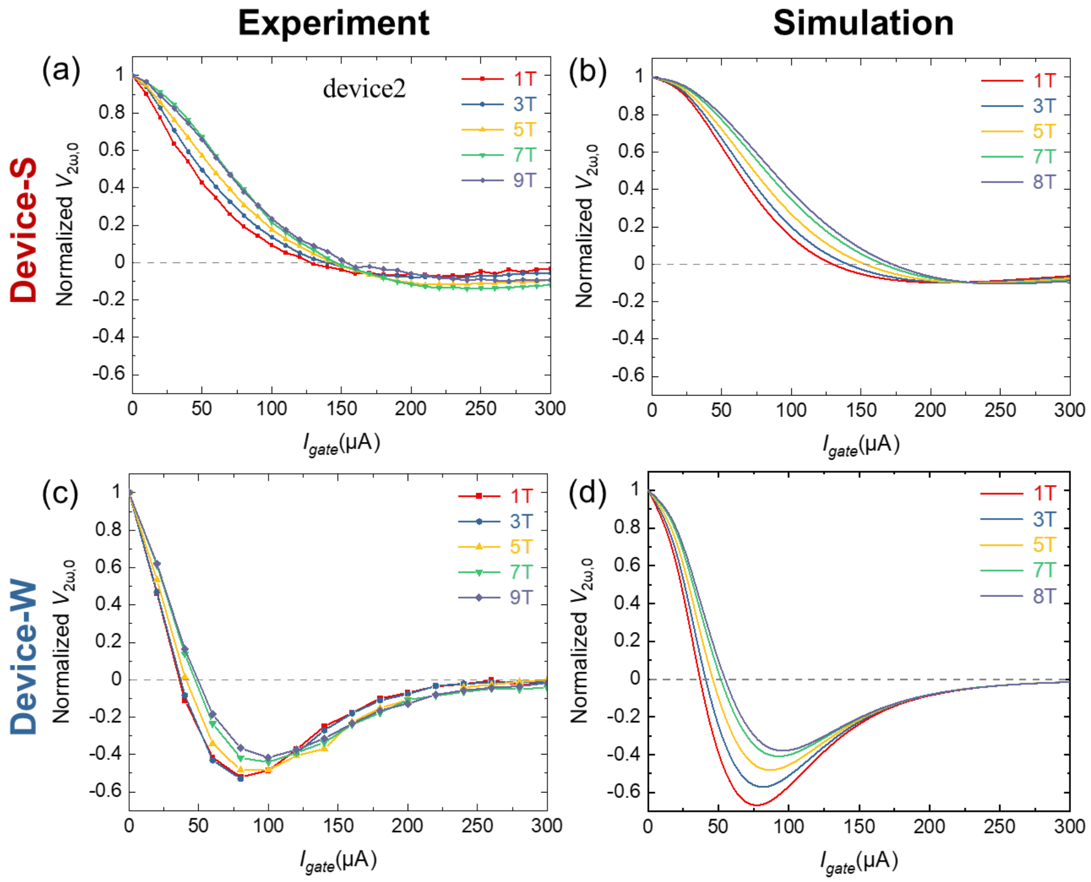

Fig. S12. I_{gate} dependent $V^S_{2\omega,0}$ and $V^W_{2\omega,0}$ under various magnetic field: Experiments and Simulations. (a) Experiment and (b) Simulation of normalized

$V^S_{2\omega,0}$ as a function of I_{gate} under multiple values of magnetic field. (c) Experiment and (d) Simulation of normalized $V^W_{2\omega,0}$ as a function of I_{gate} under multiple values of magnetic field. Only three global parameters are needed to produce all the simulation curves which match well with the experimental data.

S13. Simulation analysis for anisotropic magnon transport tuning

To further analyze the anisotropic gate tuning effect of the magnon transport in $CrPS_4$, we decompose the simulated SHM signal to S_n, ∇T components for device2 shown in Figure 3 in the main text, and display their dependence on I^2_{gate} in Fig. S13.1a. First, due to the highly anisotropic dispersion resulted by the anisotropy of magnetic exchange coupling, the band dispersion along <010> direction (with stronger magnetic coupling) has a larger group velocity $\mathbf{v} = \frac{\partial E}{\partial k}$, thus S_y for Device-S has a larger amplitude than S_x for Device-W, which has been discussed in section S8. Second, the anisotropic lattice could induce anisotropic thermal conductivity, leading to anisotropic temperature gradience in the device. With these two factors, the spin current J_m for Device-W reach to its maximum faster than Device-S with increasing the square of the gate current I^2_{gate} as shown in Fig. S13.1b. In our real-time lock-in measurement, the maximum point of J_m corresponds to the sign reversal point of $V_{2\omega,0}$, because the thermally driven magnon spin current J_m first increase and then decrease with I^2_{gate}, i.e. magnons are accumulating and flowing away below the detector electrode[10]. Thus, Device-W could be tuned faster to zero than Device-S by the DC current.

In order to test if the anisotropic exchange interaction is the dominant cause of the anisotropic magnon transport behavior, we plot another simulated $V^S_{2\omega,0}$ vs. I_{gate} curve for Device-S using the same parameters obtained from fitting the experimental $V^W_{2\omega,0}$ vs. I_{gate}curve of Device-W: $C^W = 0.484 \times 10^{-26} \mathrm{V} \cdot \mathrm{s}/\hbar$, $c^W_1 = 4.9 \times 10^{-4} \mathrm{K}/$

μA^2 and $c_2^W = 3.8 \times 10^{-4}$K/μA^2, as shown in Fig. S13.2b. Fig.13.2a is the experimental data for device 2, which is the same as shown in Fig. 3a in the main text. As can be seen in Fig. 13.2b, the simulated signal of Device-S is about four times stronger than the signal of Device-W without DC modulation, which well reproduced the experimental data. Besides, the simulation captures the most important feature of the two zero points $I_{0,1}^{S,W}$ with $I_{0,1}^{S} > I_{0,1}^{W}$. This indicates that, compared with anisotropic thermal effect, the anisotropy of the spin Seebeck coefficient $\mathbf{S}$ caused by the anisotropic in-plane magnetic exchange coupling is the main reason for the anisotropic magnon transport tuning.

That being said, the value of c_1^S is different from c_1^W, and c_2^S is different from c_2^W, in order to get the best fit to the experimental data as shown in Fig. 3a, which point to effects beyond anisotropic exchange coupling. Possible additional factors includes but not limited to anisotropic thermal conductivity, anisotropic magnon scattering rate and anisotropic phonon-magnon coupling along the <100> and <010> directions in $CrPS_4$, which are currently unknown experimentally, and warrant further investigation in future studies.

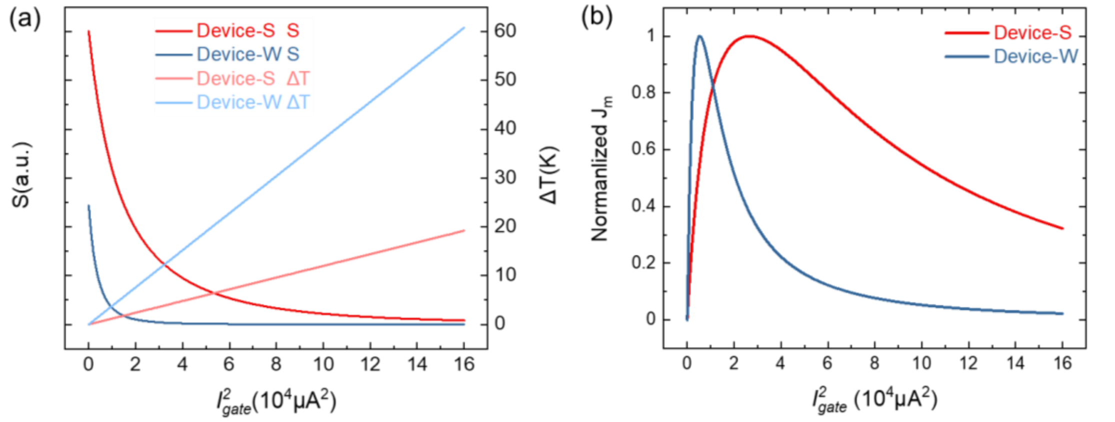

Fig. S13.1. Anisotropic Spin Seebeck effect in $CrPS_4$. (a) The simulated spin Seebeck coefficient S_n and temperature difference ΔT for Device-S (red curves) and Device-W (blue curves) as a function of the square of the gate current I_{gate}^2. (b)

The simulated normalized spin current J_m for Device-S (red curves) and Device-W (blue curves) as a function of the square of the gate current I_{gate}^2.

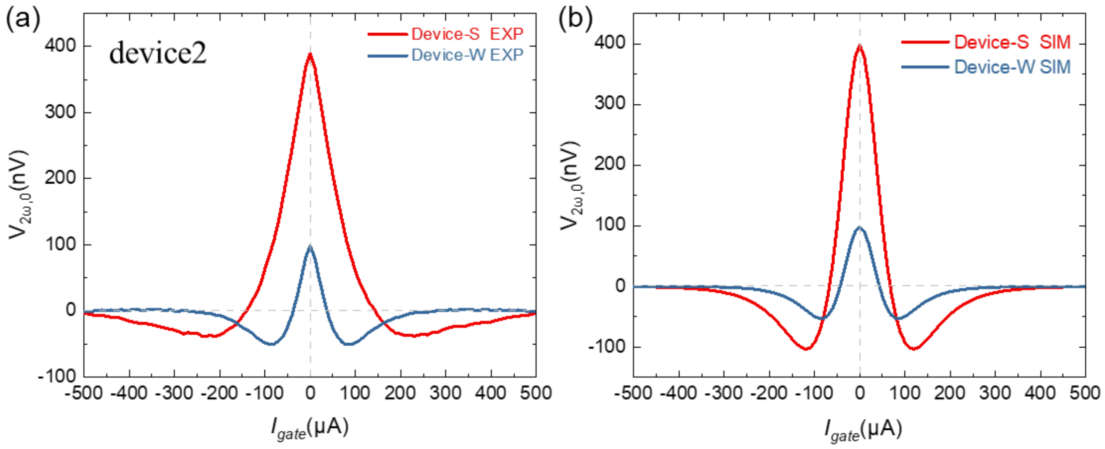

Fig. S13.2. Simulation results of DC gate tuning for $V_{2\omega,0}^{S,W}$ using the same heating efficiency parameters. (a) Experimental results of Device-S (red curve) and Device-W (blue curve), same as shown in Fig.3a in the main text. (b) The $V_{2\omega,0}^{S}$ plot of Device-S (red curve) using the same heating parameters as Device-W's simulated curve (blue curve).

S14. Stability test of few-layer $CrPS_4$ devices

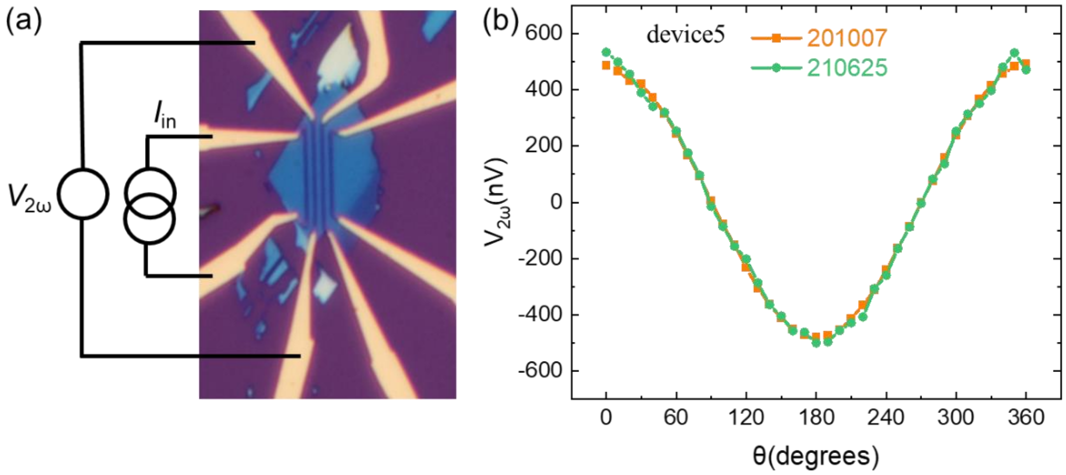

Fig. S14. Stability test of few-layer $CrPS_4$ crystals and devices. (a) The optical micrograph of few-layer $CrPS_4$ on SiO_2 substrate after exfoliation for about 9 months. (b) The device performance of our $CrPS_4$ device right after fabrication and after about

9 months.

S15. Possible applications of the magnon ROM

It is worth noting that with its peculiar readout scheme, such magnon ROMs can serve in special purposed information storage such as inscribing proprietary and confidential information. That is to say, the information stored in such magnon ROMs is out of reach for persons without a prior knowledge of several factors including the Néel temperature of the channel materials, the channel crystal orientation and magnetization direction, the preset I_{gate}, etc. The anisotropic magnon ROM could also be used to store two sets of digital information, which can be read out using two different gate currents (i.e., $I_{gate} = I_0^S$ and $I_{gate} = I_0^W$ represent two sets of information for the same ROM). Multi-state (instead of binary) memory can in principle be engineered by making use of the nonlinear and anisotropic relation between I_{gate} and I_{read} along the two directions.

References

1. Lee, J. et al. Structural and Optical Properties of Single- and Few-Layer Magnetic Semiconductor $CrPS_4$. *ACS nano* **11**, 10935-10944 (2017).
2. Joe, M., Lee, J., Lee, C. Dominant in-plane cleavage direction of $CrPS_4$. *Comput. Mater. Sci.* **162**, 277-280 (2019).
3. Liu, Y. et al. Raman Signatures of Broken Inversion Symmetry and In-Plane Anisotropy in Type-II Weyl Semimetal Candidate $TaIrTe_4$. *Adv. Mater.* **30**, e1706402 (2018).
4. Kim, S., Lee, J., Lee, C., Ryu, S. Polarized Raman Spectra and Complex Raman Tensors of Antiferromagnetic Semiconductor $CrPS_4$. *J. Phys. Chem. C* **125**, 2691-2698 (2021).
5. Liu, T. et al. Spin caloritronics in a $CrBr_3$-based magnetic van der Waals heterostructure. *Phys. Rev. B* **101**, 205407 (2020).
6. Calder, S. et al. Magnetic structure and exchange interactions in the layered semiconductor $CrPS_4$. *Phys. Rev. B* **102**, 024408 (2020).
7. Rezende, S. M., Rodríguez-Suárez, R. L., Azevedo, A. Theory of the spin Seebeck effect in antiferromagnets. *Phys. Rev. B* **93**, 014425 (2016).
8. Sanders, D. J., Walton, D. Effect of magnon-phonon thermal relaxation on heat transport by magnons. *Phys. Rev. B* **15**, 1489-1494 (1977).
9. Agrawal, M. et al. Direct measurement of magnon temperature: new insight

into magnon-phonon coupling in magnetic insulators. *Phys. Rev. Lett.* **111**, 107204 (2013).
10. Chen, G. et al. Electrically switchable van der Waals magnon valves. *Nat. Commun.* **12**, 6279 (2021).
